# The impact of comorbidities and their stacking on short- and long-term prognosis of patients over 50 with community-acquired pneumonia

**DOI:** 10.1186/s12879-021-06669-5

**Published:** 2021-09-14

**Authors:** E. Blanc, G. Chaize, S. Fievez, C. Féger, E. Herquelot, A. Vainchtock, J. F. Timsit, J. Gaillat

**Affiliations:** 1 Pfizer, Paris, France; 2 HEVA, Lyon, France; 3 EMIBiotech, Paris, France; 4 ICUREsearch, Paris, France; 5 Medical and Infectious Diseases ICU, Bichat-Claude Bernard Hospital, APHP, Paris, France; 6 UMR 1137-IAME Team 5-DeSCID: Decision SCiences in Infectious Diseases Control and Care INSERM/University of Paris, Paris, France; 7 Infectious Diseases Department, Annecy-Genevois Hospital, Annecy, France

**Keywords:** Community-acquired pneumonia, Pneumococcal pneumonia, At-risk comorbidities, High-risk comorbidities, Comorbidities stacking, Long-term mortality, Prognostic factors, Severe pneumonia, Elderly, Nonagenarians

## Abstract

**Background:**

The prognosis of patients hospitalized with community-acquired pneumonia (CAP) with regards to intensive care unit (ICU) admission, short- and long-term mortality is correlated with patient’s comorbidities. For patients hospitalized for CAP, including P-CAP, we assessed the prognostic impact of comorbidities known as at-risk (AR) or high-risk (HR) of pneumococcal CAP (P-CAP), and of the number of combined comorbidities.

**Methods:**

Data on hospitalizations for CAP among the French 50+ population were extracted from the 2014 French Information Systems Medicalization Program (PMSI), an exhaustive national hospital discharge database maintained by the French Technical Agency of Information on Hospitalization (ATIH). Their admission diagnosis, comorbidities (nature, risk type and number), other characteristics, and their subsequent hospital stays within the year following their hospitalization for CAP were analyzed. Logistic regression models were used to assess the associations between ICU transfer, short- and 1-year in-hospital mortality and all covariates.

**Results:**

From 182,858 patients, 149,555 patients aged ≥ 50 years (nonagenarians 17.8%) were hospitalized for CAP in 2014, including 8270 with P-CAP. Overall, 33.8% and 90.5% had ≥ 1 HR and ≥ 1 AR comorbidity, respectively. Cardiac diseases were the most frequent AR comorbidity (all CAP: 77.4%). Transfer in ICU occurred for 5.4% of CAP patients and 19.4% for P-CAP. Short-term and 1-year in-hospital mortality rates were 10.9% and 23% of CAP patients, respectively, significantly lower for P-CAP patients: 9.2% and 19.8% (HR 0.88 [95% CI 0.84–0.93], p < .0001). Both terms of mortality increased mostly with age, and with the number of comorbidities and combination of AR and HR comorbidities, in addition of specific comorbidities.

**Conclusions:**

Not only specific comorbidities, but also the number of combined comorbidities and the combination of AR and HR comorbidities may impact the outcome of hospitalized CAP and P-CAP patients.

**Supplementary Information:**

The online version contains supplementary material available at 10.1186/s12879-021-06669-5.

## Introduction

Community-acquired pneumonia (CAP) are a major cause of morbidity and mortality, especially in elderly [1, 2]. Thus, the associated burden increases with the ageing of population [3]. This latter is also associated with an increasing prevalence of patients with comorbidity, and furthermore multiple comorbidities [4].

The pathogens involved in CAP remain often not identified [5]. Bacteriologically confirmed CAPs involve most frequently *Streptococcus pneumoniae*, at variable rate [2, 5, 6]. Some comorbidities are recognized risk factors for pneumococcal CAP (P-CAP) occurrence and severity, i.e., short-term prognosis; those associated with an intermediate risk, generally in immunocompetent patients, are termed at risk (AR) comorbidities, while those generally associated with immunosuppression or immunodeficiency, i.e., patients with cancer or other causes of immunosuppression or immunodeficiency, are termed high risk (HR) comorbidities [7, 8].

Many studies evaluated the risk factors for the occurrence of CAP and more specifically of P-CAP. Some studies evaluated whether these factors might also be prognostic factors for short-term outcomes, e.g., transfer in ICU or short-term mortality [9–11], while the long-term mortality is less often evaluated [11–13]. However, only few studies evaluated the prognostic impact of the stacking of several comorbidities, although ageing is so often associated with multiple comorbidities [10]. This knowledge is instrumental to accurately target prevention strategies for CAP and more specifically P-CAP towards at-risk and high-risk patients.

Therefore, we extracted data from an administrative comprehensive database and conducted this study in patients aged 50 years and older and hospitalized for CAP, pneumococcal or not, to provide an accurate picture of comorbidities that are prognostic factors for in-hospital mortality during the initial hospital stay and during subsequent stays within the following year. In addition, the impact of the number, type (at risk (AR) or high-risk (HR) comorbidity), and combination of comorbidities per patient were assessed. For sake of simplicity, we used CAP for all-cause CAP, while P-CAP is used for CAP of pneumococcal origin.

## Material and methods

### Study objectives

This study aimed at evaluating the impact of comorbidities identified as risk factors for P-CAP occurrence and severity, on short-term outcomes (ICU admission, mortality during the initial hospital stay) and also on 1-year in-hospital mortality, in an exhaustive population of patients aged 50 years or more who are hospitalized in France with CAP, of pneumococcal origin or not.

Comorbidities according to the two commonly defined levels of risk of CAP or P-CAP occurrence, at risk (AR) and high risk (HR) [7, 8], i.e., comorbidities in immunocompetent and immunocompromised patients, respectively, are detailed in the Additional file 1. As one patient can present with several comorbidities that may belong to one or the other risk level, we linked the comorbidity, and not the patient, to its risk level.

### Study population

This study was conducted using data of a French clinical and administrative database, the Information Systems Medicalization Program (Programme de Médicalisation des Systèmes d’Information, PMSI) database, which records all discharges of public and private hospitals in France. Data collected on the hospital stay were the month of admission, the length of stay (days), whether the patient was admitted in ICU, and the length of the ICU stay. The main diagnosis leading to hospital admission was coded using the International Statistical Classification of Diseases, 10th Revision (ICD-10) (List of codes: see Additional file 1). A hospital admission for pneumonia was defined as an admission with a principal diagnosis of pneumonia, or a secondary diagnosis of pneumonia if the principal diagnosis was respiratory failure or sepsis. Associated diagnoses might be added in case of concomitant disease that increased the burden of care, or if it was managed during the hospital stay in addition of the management of the main diagnosis. This administrative registry enables to chain all hospital stays of individual patients.

Patients below 50 years old and with a hospital stay with a diagnosis of pneumonia within the previous 3 months were excluded.

For each initial hospital stay, the following individual de-identified data were obtained: demographics (age, gender), hospital main characteristics, patient’s comorbidities according to ICD-10 and recorded within the previous 5 years, the length of the initial hospital stay and in-hospital mortality. For each patient, the characteristics of the subsequent hospital stays within the year following the initial stay were also collected, as were the alcohol and tobacco consumptions. The 1-year in-hospital mortality was defined as death occurring at hospital during the subsequent hospital stays within the year following the initial admission for CAP. Patients were sorted out by age group: 50–69 years old, 70–89 years old, and from 90 years old. Models used the AR or HR comorbidities.

### Statistical methodology

All hospital stays were considered to be independent and included into the analyses. Standard descriptive statistics were performed for the whole cohort for the full study period. Described data were summarized as frequency and percentage for categorical variables and median and interquartile or mean and standard deviation for continuous variables.

With regards to comorbidities, the presence of at least one AR or HR comorbidities, the number of AR or HR comorbidities, the combination of AR and HR comorbidities, and the presence of each AR or HR comorbidity separately were considered.

The associations between P-CAP diagnosis and all covariates previously described, and between the transfer into ICU and all covariates previously described, were assessed using logistic regression models adjusted on age and sex. The results were expressed as odd-ratios (OR) and their 95% confidence intervals. Interactions between HR comorbidities and AR comorbidities were tested using Wald tests in logistic models with an interaction term between AR and HR comorbidities. They were all adjusted on age and sex. In addition, the association between occurrence of short-term and 1-year in-hospital death and all covariates previously described were assessed using Cox models adjusted on sex and age.

P-values less than 0.05 were considered significant. All statistical analyses were performed using the SAS software (version 9.4; Cary, NC, USA).

## Results

### Study population

Of a total of 182,858 patients hospitalized for CAP (i.e., all-cause CAP) in France in 2014 and without any hospitalization for pneumonia within the previous 3 months, those aged at least 50 years were 149,555 (81.8%). From these, a subset of 8270 (5.5%) patients had a P-CAP (Additional file 1: Fig. S1). As the French population over 50 years of age was 23,788,401 in 2014, the national incidence of the hospitalizations for CAP and P-CAP this year reached 629 and 34.8 per 100,000 populations, respectively.

The main characteristics of patients with CAP and more specifically P-CAP are depicted in Table 1. The median age of patients with P-CAP was 73.7 years while those with CAP were aged 78.1 years. The median length of stay of patients with all-causes CAP or more specifically P-CAP was 8 [4-13] and 9 [6-15] days, respectively.

**Table 1:** Characteristics of patients > 50 years old hospitalized for community-acquired pneumonia all cause, overall and by age group, and for pneumococcal community-acquired pneumonia

Characteristics of the patients (n (% of overall))	CAP > 50 y.o. (149,555 (100%))	P-CAP > 50 y.o. (8270 (5.53%))	CAP 50–69 y.o. (37,807(25.3%))	CAP 70–89 y.o. (85,159 (56.9%))	CAP ≥ 90 y.o. (26,589 (17.8%))
Male gender	78,862 (52.7%)	4794 (58.0%)	23,510 (62.2%)	45,753 (53.7%)	9599 (36.1%)
Age (years)	78.1 (12.0)	73.7 (12.4)	60.9 (5.5)	81.2 (5.4)	92.9 (2.8)
Alcohol	12,614 (8.4%)	1187 (14.4%)	6,980 (18.5%)	5234 (6.15%)	400 (1.50%)
Tobacco	18,225 (12.2%)	1912 (23.1%)	10,151 (26.8%)	7530 (8.84%)	544 (2.05%)
No comorbidity	10,847 (7.3%)	570 (6.89%)	6,333 (16.8%)	3620 (4.25%)	894 (3.36%)
*HR comorbidities*	50,587 (33.8%)	2962 (35.8%)	14,137 (36.8%)	29,732 (34.9%)	6718 (25.3%)
Solely HR comorbidities	3349 (2.24%)	201 (2.4%)	1898 (5.02%)	1301 (1.53%)	150 (0.56%)
No HR comorbidity	98,968 (66.2%)	5308 (64.2%)	23,670 (62.6%)	55,427 (65.1%)	19,871 (74.7%)
One HR comorbidity	42,670 (28.5%)	2423 (29.3%)	11,432 (30.2%)	25,203 (29.6%)	6035 (22.7%)
≥ 2 HR comorbidities	7917 (5.29%)	539 (6.5%)	2705 (7.15%)	4,529 (5.32%)	683 (2.57%)
Number of HR comorbidities	0.40 (0.62)	0.44 (0.66)	0.47 (0.69)	0.41 (0.62)	0.28 (0.51)
Solid tumor	36,540 (24.4%)	2042 (24.7%)	9885 (26.2%)	21,717 (25.5%)	4938 (18.6%)
Hematological malignancy	10,693 (7.15%)	705 (8.52%)	2491 (6.59%)	6736 (7.91%)	1466 (5.51%)
Auto-immune disorders	6731 (4.50%)	384 (4.64%)	1860 (4.92%)	4113 (4.83%)	758 (2.85%)
Primitive immune deficiency	2392 (1.60%)	187 (2.26%)	957 (2.53%)	1276 (1.50%)	159 (0.60%)
Organ transplant recipient	2298 (1.54%)	153 (1.85%)	1567 (4.14%)	654 (0.77%)	77 (0.29%)
HIV	698 (0.47%)	91 (1.10%)	578 (1.53%)	118 (0.14%)	2 (0.01%)
Asplenia—hyposplenia	636 (0.43%)	705 (8.52%)	266 (0.70%)	324 (0.38%)	46 (0.17%)
*AR comorbidities*	135,359 (90.5%)	7499 (90.7%)	29,576 (78.2%)	80,238 (94.2%)	25,545 (96.1%)
Solely AR comorbidities	88,121 (58.9%)	4738 (57.3%)	17,337 (45.9%)	51,807 (60.8%)	18,977 (71.4%)
No AR comorbidity	14,196 (9.5%)	771 (9.32%)	8231 (21.8%)	4921 (5.78%)	1044 (3.93%)
One AR comorbidity	32,150 (21.5%)	1819 (22.0%)	10,547 (27.9%)	16,646 (19.5%)	4957 (18.6%)
2 AR comorbidities	41,724 (27.9%)	2537 (30.7%)	9295 (24.6%)	24,531 (28.8%)	7898 (29.7%)
3 AR comorbidities	33,826 (22.6%)	1862 (22.5%)	5802 (15.3%)	20,976 (24.6%)	7048 (26.5%)
≥ 4 AR comorbidities	27,659 (18.5%)	1281 (15.5%)	3932 (10.4%)	18,085 (21.2%)	5642 (21.2%)
Number of AR comorbidities	2.27 (1.39)	2.19 (1.32)	1.69 (1.36)	2.45 (1.35)	2.51 (1.28)
Chronic cardiac diseases	115,702 (77.4%)	6118 (74.0%)	21,630 (57%)	70,749 (83.1%)	23,323 (87.7%)
Malnutrition	52,338 (35.0%)	2651 (32.1%)	8819 (23.3%)	30,732 (36.1%)	12,787 (48.1%)
Chronic respiratory diseases	45,696 (30.6%)	3782 (45.7%)	12,710 (33.6%)	27,124 (31.9%)	5862 (22.1%)
Diabetes mellitus	35,250 (23.6%)	1905 (23.0%)	8067 (21.3%)	22,917 (26.9%)	4266 (16.0%)
Neurodegenerative diseases	30,027 (20.1%)	848 (10.3%)	1929 (5.10%)	20,246 (23.8%)	7852 (29.5%)
Chronic kidney diseases	28,074 (18.8%)	1281 (15.5%)	3832 (10.1%)	17,271 (20.3%)	6971 (26.2%)
Stroke	24,605 (16.5%)	874 (10.6%)	3367 (8.9%)	16,187 (19.0%)	5051 (19.0%)
Chronic liver diseases	7599 (5.08%)	651 (7.87%)	3466 (9.2%)	3631 (4.26%)	502 (1.89%)
*HR and AR comorbidities*					
≥ 1 HR and ≥ 1 AR	47,238 (31.6%)	2761 (33.4%)	12,239 (32.4%)	28,431 (33.4%)	6568 (24.7%)

*CAP* community-acquired pneumonia, *P-CAP* pneumococcal community-acquired pneumonia, *HR* high-risk comorbidities (immunodepression, immunodeficiency, cancer), *AR* at risk comorbidities (comorbidities at risk of pneumococcal CAP in immunocompetent patients), *HIV* human immunodeficiency virus, *Q1–Q3* interquartile range. Qualitative variables are expressed as number of patients (percentages), quantitative variables as mean (SD)

### Prevalence of comorbidities

Among all patients with CAP, and similarly for those with P-CAP, solely a small minority had no comorbidity (10,847 (7.2%) and 570 (6.9%), respectively), and even less had solely HR comorbidities (3,349 (2.2%) and 201 (2.4%), respectively) (Table 1). Except for the youngest patients, more than half of the CAP patients have at least one AR comorbidity; most often cardiac diseases, followed by malnutrition and respiratory chronic diseases (all CAP, 77.4%, 35.0% and 30.6%, respectively). Of note, the cardiac diseases category included hypertension, essential or not. Solid tumor was the most frequent HR comorbidities (all CAP, 24.4%). The comorbidities profile of P-CAP patients was not so different, as described in Table 2.

**Table 2:** Comorbidity or other characteristics that are risk factors for pneumococcal pneumonia among patients > 50 years old hospitalized for community-acquired pneumonia, after adjustment on age and sex

Characteristics of the patients	All patients		Age group 50–69 y.o.		Age group 70–89 y.o		Age group ≥ 90 y.o.	
	OR [95% CI]	p value	OR [95% CI]	p value	OR [95% CI]	p value	OR [95% CI]	p value
Alcohol abuse	1.48 [1.38;1.58]	< 0.0001	1.64 [1.50;1.78]	< 0.0001	1.25 [1.11;1.41]	0.0002	1.39 [0.87;2.23]	0.163
Tobacco abuse	1.83 [1.73;1.94]	< 0.0001	1.79 [1.66;1.93]	< 0.0001	1.88 [1.72;2.06]	< 0.0001	2.21 [1.58;3.10]	< 0.0001
*HR comorbidities*	1.03 [0.99;1.08]	0.17	0.94 [0.87;1.01]	0.1	1.11 [1.04;1.19]	0.001		
No HR comorbidity	1.00 (ref)		1.00 (ref)		1.00 (ref)		1.00 (ref)	
N = 1 HR comorbidity	1.01 [0.96;1.06]	0.67	0.93 [0.86;1.01]	0.08	1.08 [1.01;1.16]	0.024	0.97 [0.82;1.14]	0.6855
N ≥ 2 HR comorbidities	1.15 [1.05;1.26]	0.003	0.98 [0.85;1.13]	0.76	1.30 [1.14;1.47]	< 0.0001	1.39 [0.97;2.00]	0.0766
*Type of HR comorbidities*								
Solid tumor	0.96 [0.91;1.02]	0.16	0.90 [0.83;0.98]	0.0158	1.02 [0.95;1.10]	0.5511	0.90 [0.75;1.07]	0.2417
Hematological malignancy	1.22 [1.13;1.33]	< 0.0001	1.06 [0.92;1.22]	0.4225	1.30 [1.17;1.45]	< 0.0001	1.39 [1.08;1.79]	0.0116
Auto-immune disorders	1.01 [0.91;1.12]	0.87	0.89 [0.75;1.06]	0.1962	1.09 [0.95;1.26]	0.2381	1.10 [0.75;1.62]	0.6156
Primitive immune deficiency	1.28 [1.10;1.49]	0.001	1.15 [0.92;1.43]	0.2130	1.42 [1.13;1.77]	0.0022	1.71 [0.87;3.36]	0.1207
Organ transplant recipient	0.89 [0.75;1.05]	0.16	0.81 [0.67;0.99]	0.0391	1.20 [0.86;1.67]	0.2792	0.76 [0.19;3.11]	0.7055
HIV	1.72 [1.38;2.15]	< 0.0001	1.73 [1.36;2.19]	< 0.0001	1.77 [0.92;3.38]	0.0849	–	–
Asplenia—hyposplenia	1.21 [0.90;1.63]	0.204	1.40 [0.96;2.05]	0.0766	0.82 [0.47;1.42]	0.4733	2.69 [0.96;7.53]	0.0588
*AR comorbidities*	1.35 [1.25;1.46]	< 0.0001	1.53 [1.39;1.69]	< 0.0001	1.06 [0.92;1.21]	0.44		
No AR comorbidity	1.00 (ref)		1.00 (ref)		1.00 (ref)		1.00 (ref)	
N = 1 AR comorbidity	1.26 [1.15;1.38]	< 0.0001	1.33 [1.19;1.49]	< 0.0001	1.05 [0.90;1.22]	0.54	1.04 [0.72;1.52]	0.8294
N = 2 AR comorbidities	1.49 [1.36;1.62]	< 0.0001	1.62 [1.44;1.81]	< 0.0001	1.20 [1.04;1.38]	0.02	1.16 [0.81;1.67]	0.4162
N = 3 AR comorbidities	1.40 [1.28;1.53]	< 0.0001	1.75 [1.55;1.98]	< 0.0001	1.06 [0.92;1.23]	0.41	1.00 [0.69;1.44]	0.9949
N ≥ 4 AR comorbidities	1.19 [1.08;1.31]	< 0.00001	1.60 [1.39;1.84]	< 0.0001	0.87 [0.74;1.01]	0.07	0.92 [0.63;1.33]	0.6542
*Type of AR comorbidities*								
Chronic cardiac diseases	1.03 [0.98;1.09]	0.26	1.07 [0.99;1.15]	0.0775	0.98 [0.90;1.06]	0.6197	1.09 [0.88;1.34]	0.4324
Malnutrition	0.98 [0.94;1.03]	0.54	1.29 [1.19;1.40]	< .0001	0.82 [0.77;0.88]	< 0.0001	1.03 [0.90;1.18]	0.6242
Chronic respiratory diseases	1.91 [1.83;2.00]	< 0.0001	2.04 [1.90;2.20]	< .0001	1.94 [1.82;2.06]	< 0.0001	1.38 [1.19;1.60]	< 0.0001
Diabetes mellitus	0.96 [0.91;1.02]	0.16	1.02 [0.93;1.11]	0.6883	0.96 [0.89;1.03]	0.2403	0.76 [0.63;0.93]	0.0079
Neurodegenerative diseases	0.52 [0.49;0.56]	< 0.0001	0.56 [0.46;0.69]	< 0.0001	0.48 [0.44;0.53]	< 0.0001	0.68 [0.58;0.80]	< 0.0001
Chronic kidney diseases	0.89 [0.83;0.94]	0.0001	0.88 [0.77;0.99]	0.0380	0.86 [0.79;0.93]	0.0003	1.00 [0.86;1.16]	0.9677
Stroke	0.64 [0.60;0.69]	< 0.0001	0.70 [0.61;0.81]	< 0.0001	0.60 [0.55;0.66]	< 0.0001	0.74 [0.61;0.89]	0.0012
Chronic liver diseases	1.38 [1.27;1.50]	< 0.0001	1.45 [1.30;1.62]	< 0.0001	1.35 [1.18;1.55]	< 0.0001	0.81 [0.47;1.38]	0.4372

*CAP* community-acquired pneumonia, *P-CAP* pneumococcal community-acquired pneumonia, *HR* high-risk comorbidities (immunodepression, immunodeficiency, cancer), *AR* at risk comorbidities (comorbidities at risk of pneumococcal CAP in immunocompetent patients), *y.o.* year old, *HIV* human immunodeficiency virus, *OR* odds ratio, *CI* confidence interval

Data on the comorbidities according to the age group are detailed in Tables 1 and 2. Patients with solely HR comorbidities were less frequent when age increased, while it was the contrary for AR comorbidities. AR comorbidities increased with the age of patients, both in terms of prevalence among the patients, and in terms of number per individual patients.

### Outcome according to the comorbidities and age

**Fig. 1:**
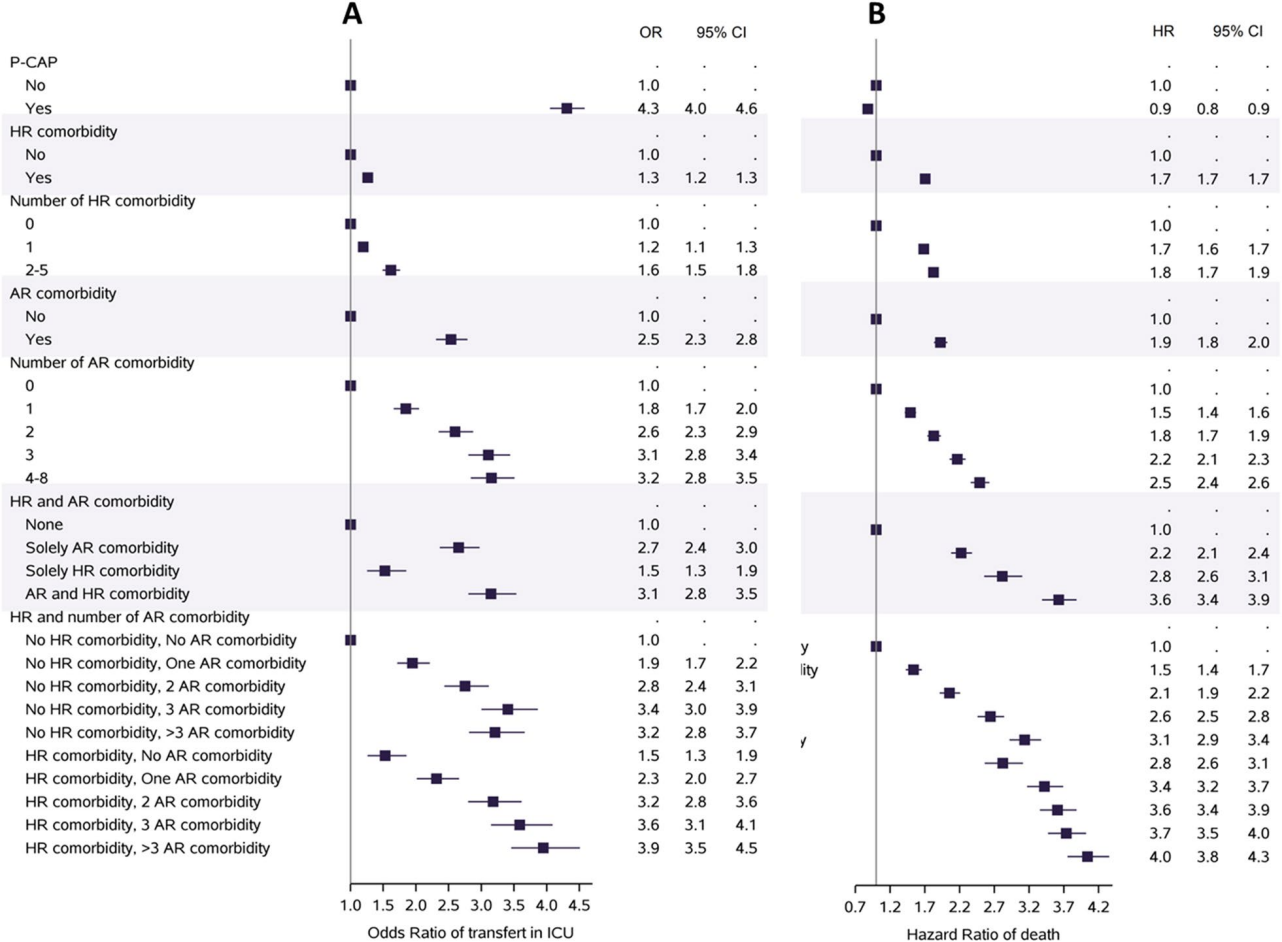
Impact of the accumulation of comorbidities according to their level of associated risk (high-risk (HR) or at-risk (AR)) on the 1-year in-hospital mortality of patients with community-acquired pneumonia; **A** transfer into intensive care unit; **B** 1-year in-hospital mortality. *CAP* community-acquired pneumonia, *P-CAP* pneumococcal community-acquired pneumonia, *HR* high-risk comorbidities (immunodepression, immunodeficiency, cancer), *AR* at risk comorbidities (comorbidities at risk of pneumococcal CAP in immunocompetent patients), *ICU* intensive care unit, *HR* hazard ratio, *95% CI* 95% confidence interval

The proportion of patients hospitalized for CAP and requiring to be admitted in ICU was 5.4% (8095/149,555), while 19.4% (1608/8270) patients with P-CAP required an ICU admission (adjusted odds ratio (aOR) 4.31, 95% CI [4.05–4.58], p < 0.0001) (Fig. 1 and Additional file 1: Table S2). CAP patients were less often admitted to ICU if they were older (Odds Ratio (OR) 0.07, 95% CI [0.06–0.08], p < 0.0001), and with comorbidities, HR and AR, and even less often if they cumulated several AR comorbidities (Fig. 1A, and Additional file 1: Table S3).

Among patients with CAP, the in-hospital mortality during the initial stay reached 10.9%. It increased with age, from 6.0 to 11.3% and 16.8% in the three age groups of patients with CAP, respectively (Additional file 1: Table S4). The subset of patients with P-CAP had a significantly lower early mortality (9.2% vs. 11.0%, hazard ratio (HR) 0.650, 95%CI [0.604–0.699], p < 0.001).

The all-cause 1-year in-hospital mortality rate, i.e., within the year following the hospitalization including the initial stay, was 23.0% and 19.8% for patients hospitalized with CAP and P-CAP, respectively, leading to an annual national in-hospital mortality incidence of 145 and 6.9 per 100,000 populations, respectively. The mortality rate increased with age, from 16.9 to 23.9% and 28.9% in the three age groups of patients with CAP, respectively. The subset of patients with P-CAP had a significantly lower 1-year in-hospital mortality rate (HR 0.88 (95% CI [0.84–0.93], p < 0.0001) (Additional file 1: Table S5).

**Fig. 2:**
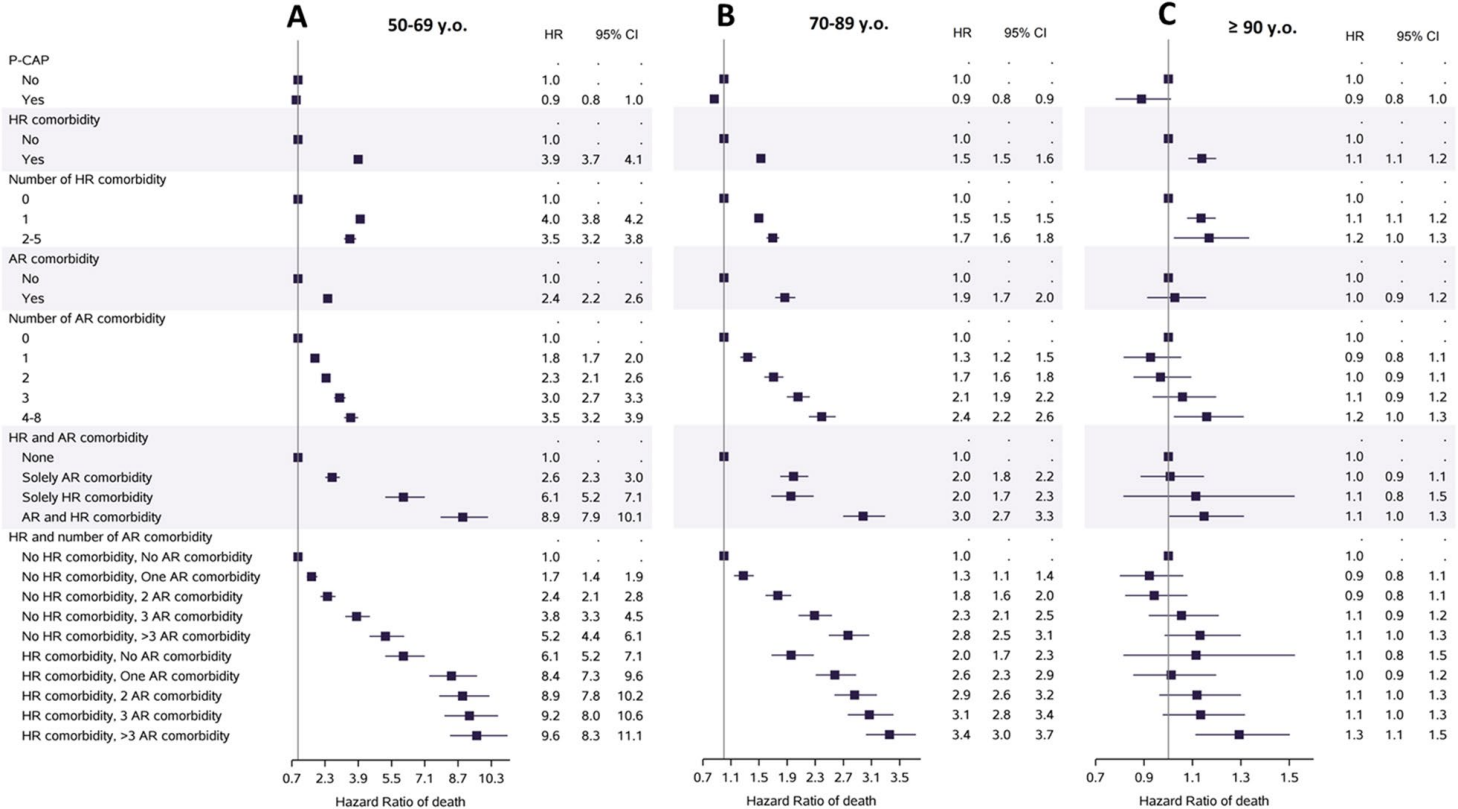
Impact of the accumulation of comorbidities according to their level of associated risk (high-risk or at-risk) on the 1-year in-hospital mortality of patients with CAP, according to their age group, **A** 50–69 years old, **B** 70–89 years old, or **C** 90 and more years old. *CAP* community-acquired pneumonia, *P-CAP* pneumococcal community-acquired pneumonia, *HR comorbidity* high-risk comorbidities (immunodepression, immunodeficiency, cancer), *AR comorbidity* at risk comorbidities (comorbidities at risk of pneumococcal CAP in immunocompetent patients), *HR* hazard ratio

Patients with HR comorbidities had an increased mortality rate, for CAP as for P-CAP, and patients aged 50–69 years had the highest risk (HR 3.89, 95% CI [3.69–4.10], p < 0.0001) (Fig. 2). Among those patients with HR comorbidities, the 1-year mortality risk was significantly increased, and the highest risk was for those with solid tumor or hematological malignancies (HR 1.80, 95% CI [1.76–1.84], p < 0.0001, and 1.44, 95% CI [1.39–1.49], p < 0.0001, respectively) (Additional file 1: Table S6). The mortality risk increased with the number of cumulated HR comorbidities and with the number of AR comorbidities (Fig. 1B and Additional file 1: Table S6). The mortality risk was also increased for patients with AR. Those with HR and AR comorbidities had a marked increase of their mortality rate, especially in the age group 50–69 years (HR 8.93, 95% CI [7.86–10.13], p < 0.0001). Among AR comorbidities, each of them except diabetes was associated with a significantly increased mortality rate, with Hazard Ratio ranging from 1.58 for malnutrition to 1.04 for chronic respiratory diseases. Among the oldest patients, patients with AR comorbidities did not have a higher mortality risk.

## Discussion

This very large study, based on a comprehensive registry of all hospital admissions for CAP in France over one year, assessed the prognostic impact of risk factors usually more associated with the occurrence of CAP, and more specifically P-CAP, in patients aged 50 and older. These risk factors, sorted in at-risk (AR) comorbidities (generally in immunocompetent patients) and high-risk (HR) comorbidities (generally in immunocompromised patients) were frequent: overall, almost all patients had at least one AR comorbidity, and one out of three had at least one HR comorbidity. Interestingly, the short- and long-term prognosis in terms of in-hospital mortality was not only impacted by the age and by specific comorbidities, but also by the number of stacked comorbidities, and by the combination of AR and HR comorbidities.

This is of utmost importance in an ageing population, as comorbidities are more and more stacking with age increase. Of note, our population of patients with CAP comprised almost 18% nonagenarians and older, similar to that observed in other reports [14, 15]. Other studies showed the increased risk of complications and all-cause deaths of elderly patients compared to the younger ones [10, 15–18], and more specifically for patients older than 80 years compared to younger ones [10, 15]. We reported a higher 1-year in-hospital mortality rate for CAP patients versus those with P-CAP. A recent study among adult US patients requiring hospitalization for CAP reported a higher 1-year mortality rate, 30.6%, [11] compared to our study, likely because it was not limited to the in-hospital mortality.

We observed an increase of the in-hospital mortality rate with the number of comorbidities, AR or HR comorbidities. The risk was very high when AR comorbidities were combined with HR comorbidities. Of note, the prognostic impact of the number of comorbidities is rarely reported in the literature. In addition, mortality rates are different across countries and their respective Health systems and managements strategies, making any comparison delicate. While a recent Chinese study did not evidence any association between the 60-day mortality and the number of comorbidities (regardless of the type of comorbidities) [15], a higher impact of cumulated risk factors on the 30-day mortality was reported in a US study conducted among Veterans aged 50 and older with pneumococcal infection [19]. The Odds ratio increased from 2.01 (95%CI [1.47–2.75]) for patients with 2 risk factors up to 4.23 (95% CI [2.69–6.65]) for those who stacked 6 risk factors. A study conducted in Spain in adult patients with CAP and using an administrative database showed an increase of mortality with the number of comorbidities (OR 1.35 (95% CI, 1.33–1.38) for patients with ≥ 2 comorbidities versus those with none) [6]. In our study, patients with CAP, including P-CAP, have almost all at least one comorbidity and very often several comorbidities. More than half of patients had only AR comorbidities, and this proportion increased with age, as did the mean number of combined AR comorbidities per patient. Importantly, one patient out of three combined AR and HR comorbidities, while patients with only HR comorbidities were rather rare, and rarer with age. The increased risk of mortality associated with age is furthermore increased by the number of comorbidities that expectedly increases with age, although less in nonagenarians [20, 21]. Therefore, the identification of comorbidities of poor prognosis for patients with CAP is instrumental for an improved management of these patients.

An increased risk of invasive pneumococcal infection was already reported in cancer patients [22, 23]. Our results of higher incidence of pneumococcal infection and of poorer prognosis among cancer patients are of utmost importance, as therapeutic advances in cancer management itself continue to progress and the number of cancer survivors is more and more increasing.

In our study, the AR comorbidities associated with the highest risk of 1-year in-hospital mortality were chronic liver diseases, in CAP and P-CAP patients, particularly in the youngest patients. Malnutrition had also a strong impact on the 1-year mortality risk, as already shown in other studies [13]. Malnutrition may be a complication of other comorbidities such as malignant diseases or chronic liver diseases, or ageing. Importantly, the impact of these comorbidities on the 1-year in-hospital mortality is the strongest for patients aged 50–69 years, an age group with a high incidence of malignant diseases. The prognosis risk specifically associated with cardiac diseases in patients with CAP was already underlined in other studies [10, 18]. The increase of long-term mortality by cardiac complications, involved in one-third of the late-onset deaths, was also reported by others [17, 24]. Of note, our study included arterial hypertension in chronic cardiac diseases.

Given the high incidence of CAP and of P-CAP, their appropriate management, including prevention, is instrumental. P-CAP might be prevented by pneumococcal vaccination. However, studies conducted in France reported a low rate of vaccinated patients among those aged 65 years and older, around 10 to 17% [25–27], increasing to 27% (95% CI 21–34) among elderly patients with targeted comorbidities [27]. The recommendations in France at the time of this study were driven by comorbidities, and malignant diseases are listed as HR comorbidity. The increased mortality risk of CAP patients with cancer has already been described [28]. A low rate of vaccinated patients was reported by few studies specifically focused on cancer patients, from 10.1 (95% CI 4.1–16), to 39% (95% CI 33–44) [29]. Healthcare professionals and cancer societies are advocating for increasing the vaccination rate, and some are initiating efficient targeted vaccination programs [30]. The recently revised French recommendations for pneumococcal vaccination do not make any differences anymore between AR and HR comorbidities, consistently with our findings on the impact of these comorbidities on ICU admission and on mortality [31].

This study is unique, as it is a comprehensive collection of all patients hospitalized with CAP in 2014 in France, able to collect all subsequent hospital stays of a patient within the year following his hospital admission. In addition, we were able to ascertain not only the role of each comorbidity, but also the impact of the type of comorbidities, AR or HR, and of their cumulated number, which is rarely assessed.

Nevertheless, this study has also some limitations. We worked on an administrative database, and disease coding is not always very accurate, particularly for CAP [32, 33]. In addition, P-CAP are likely under-reported [34]. However, we used the ICD-10th version of the coding system, which showed more reliability for pneumonia diagnosis, and we combined codes for an improved accuracy [35, 36]. Also, coding rules specify that events occurring during the hospital stay cannot be coded as admission diagnosis; therefore, nosocomial pneumonia should not be included in our study. Second, this administrative database did not provide data on the mortality outside of the hospital. A French study showed that the rate of deaths that occurred at hospital in 2008 ranged around 60% for the patients aged between 40 and 89 years [37]. In addition, the death occurred more frequently at hospital when it was due to pneumonia. These rates were lower among the oldest patients: while more than 60% of the patients younger than 79 died at the hospital, this rate was only 58% among the octogenarians, and dropped to 42% for the nonagenarians. With regards to comorbidities, essential hypertension was included in the cardiac and vascular comorbidities, which is not consensual. In addition, some data were not available, such as data enabling to grade pneumonia severity, immunization status of the patients, microbiological characteristics of the causative strain, or comorbidities severity. Finally, we did not collect data on a reference population in order to evaluate the over-risk of 1-year in-hospital mortality related to the hospitalization for CAP or P-CAP.

## Conclusion

The burden of hospitalized CAP, and more specifically P-CAP, is associated to the short-term and long-term prognosis of these patients. This study covering all CAPs hospitalized in France over one year showed that age, and also comorbidities by themselves, are worsening the short- and long-term prognosis of these patients. Importantly, the prognosis is also worsening according to the stacking of comorbidities, and to the combination of comorbidities of medium and high-risk level. Patients with CAP and high-risk comorbidities were more likely transferred to ICU, and this risk increased with the number of stacked comorbidities. Some comorbidities have a strong impact on the 1-year mortality, such as malnutrition, or solid tumors, particularly for those aged 50–69 years, or the stacking of several AR comorbidities, which pleads for increased consideration of these conditions in CAP patients. Of note, nonagenarians are an exception, as their mortality increase seems to be more related to their age itself, rather than to the number of comorbidities.

For optimizing the long-term outcome of patients with CAP, including those with P-CAP, our findings warrant to pay more attention to patients who have some specific comorbidities, such as solid tumors, malnutrition, or chronic liver disease, and, importantly, also to patients who stack several comorbidities putting them at high risk of heavier long-term disease burden.

## Supplementary Information

**Additional file 1: Figure S1.** Patients flow chart. CAP: community-acquired pneumonia (all causes); P-CAP: community-acquired pneumococcal pneumonia; *S. pneumoniae: Streptococcus pneumoniae*; ICU: intensive care unit. The short-term deaths are those occurring during the hospital stay related to the CAP management, while the one-year in-hospital deaths are the in-hospital deaths occurring within the year following the initial hospital stay. **Table S1.** Comorbidities and other characteristics of patients with community-acquired pneumonia of all causes and of those with pneumococcal community-acquired pneumonia, according to the age groups. **Table S2.** Comorbidities or other characteristics that are risk factors for the admission in intensive care unit for patients with community-acquired pneumonia and those with pneumococcal community-acquired pneumonia, after adjustment on age and sex (Cox univariate model). **Table S3.** Comorbidity or other characteristics that are risk factors for the transfer in intensive care unit of patients > 50 years old hospitalized for community-acquired pneumonia overall, after adjustment on age and sex, overall and according to their age group (Cox univariate model). **Table S4.** In-hospital mortality of patients with community-acquired pneumonia according to the comorbidities categories, overall and according to their age group, during the initial hospital stay and during the subsequent hospital stays within the year following the CAP onset. **Table S5.** Comorbidity or other characteristics that are risk factors for the one-year in-hospital mortality for patients with community-acquired pneumonia and those with pneumococcal community-acquired pneumonia, after adjustment on age and sex (Cox univariate model). **Table S6.** Comorbidity or other characteristics that are risk factors for the one-year mortality of patients > 50 years old hospitalized for community-acquired pneumonia, after adjustment on age and sex, overall and according to their age group (Cox univariate model).

## Data Availability

The datasets used and/or analyzed during the current study are available from Pfizer’s authors upon reasonable request.

## References

[1] Divino V, Schranz J, Early M, Shah H, Jiang M, DeKoven M (2019). The annual economic burden among patients hospitalized for community-acquired pneumonia (CAP): a retrospective US cohort study. Curr Med Res Opin.

[2] Torres A, Cilloniz C, Blasi F, Chalmers JD, Gaillat J, Dartois N, Schmitt HJ, Welte T (2018). Burden of pneumococcal community-acquired pneumonia in adults across Europe: a literature review. Respir Med.

[3] Demographic balance sheet 2018. https://www.insee.fr/en/statistiques/2382601?sommaire=2382613.

[4] Wroe PC, Finkelstein JA, Ray GT, Linder JA, Johnson KM, Rifas-Shiman S, Moore MR, Huang SS (2012). Aging population and future burden of pneumococcal pneumonia in the United States. J Infect Dis.

[5] Cilloniz C, Ewig S, Gabarrus A, Ferrer M, Puig de la Bella Casa J, Mensa J, Torres A (2017). Seasonality of pathogens causing community-acquired pneumonia. Respirology.

[6] de Miguel-Diez J, Jimenez-Garcia R, Hernandez-Barrera V, Jimenez-Trujillo I, de Miguel-Yanes JM, Mendez-Bailon M, Lopez-de-Andres A (2017). Trends in hospitalizations for community-acquired pneumonia in Spain: 2004 to 2013. Eur J Intern Med.

[7] Use of 13-valent pneumococcal conjugate vaccine and 23-valent pneumococcal polysaccharide vaccine for adults with immunocompromising conditions: recommendations of the Advisory Committee on Immunization Practices (ACIP). MMWR Morb Mortal Wkly Rep. 2012;61(40):816–819. 23051612

[8] Recommendations with regards to the vaccinations for the prevention of pneumococcal infections in adults. https://www.hcsp.fr/Explore.cgi/avisrapportsdomaine?clefr=614.

[9] Waterer GW, Self WH, Courtney DM, Grijalva CG, Balk RA, Girard TD, Fakhran SS, Trabue C, McNabb P, Anderson EJ (2018). In-hospital deaths among adults with community-acquired pneumonia. Chest.

[10] Luna CM, Palma I, Niederman MS, Membriani E, Giovini V, Wiemken TL, Peyrani P, Ramirez J (2016). The impact of age and comorbidities on the mortality of patients of different age groups admitted with community-acquired pneumonia. Ann Am Thorac Soc.

[11] Ramirez JA, Wiemken TL, Peyrani P, Arnold FW, Kelley R, Mattingly WA, Nakamatsu R, Pena S, Guinn BE, Furmanek SP (2017). Adults hospitalized with pneumonia in the United States: incidence, epidemiology, and mortality. Clin Infect Dis.

[12] Kaplan V, Clermont G, Griffin MF, Kasal J, Watson RS, Linde-Zwirble WT, Angus DC (2003). Pneumonia: still the old man's friend?. Arch Intern Med.

[13] Mortensen EM, Kapoor WN, Chang CC, Fine MJ (2003). Assessment of mortality after long-term follow-up of patients with community-acquired pneumonia. Clin Infect Dis.

[14] Laporte L, Hermetet C, Jouan Y, Gaborit C, Rouve E, Shea KM, Si-Tahar M, Dequin PF, Grammatico-Guillon L, Guillon A (2018). Ten-year trends in intensive care admissions for respiratory infections in the elderly. Ann Intensive Care.

[15] Han X, Zhou F, Li H, Xing X, Chen L, Wang Y, Zhang C, Liu X, Suo L, Wang J (2018). Effects of age, comorbidity and adherence to current antimicrobial guidelines on mortality in hospitalized elderly patients with community-acquired pneumonia. BMC Infect Dis.

[16] Cillóniz C, Dominedò C, Pericàs JM, Rodriguez-Hurtado D, Torres A (2020). Community-acquired pneumonia in critically ill very old patients: a growing problem. Eur Respir Rev.

[17] Johnstone J, Eurich DT, Majumdar SR, Jin Y, Marrie TJ (2008). Long-term morbidity and mortality after hospitalization with community-acquired pneumonia: a population-based cohort study. Medicine.

[18] Hespanhol VP, Bárbara C. Pneumonia mortality, comorbidities matter? Pulmonology. 2019;S2531-0437(2519)30205-30203. 10.1016/j.pulmoe.2019.10.003 31787563

[19] Morton JB, Morrill HJ, LaPlante KL, Caffrey AR (2017). Risk stacking of pneumococcal vaccination indications increases mortality in unvaccinated adults with *Streptococcus pneumoniae* infections. Vaccine.

[20] Prados-Torres A, Poblador-Plou B, Calderon-Larranaga A, Gimeno-Feliu LA, Gonzalez-Rubio F, Poncel-Falco A, Sicras-Mainar A, Alcala-Nalvaiz JT (2012). Multimorbidity patterns in primary care: interactions among chronic diseases using factor analysis. PLoS ONE.

[21] van Oostrom SH, Picavet HS, van Gelder BM, Lemmens LC, Hoeymans N, van Dijk CE, Verheij RA, Schellevis FG, Baan CA (2012). Multimorbidity and comorbidity in the Dutch population—data from general practices. BMC Public Health.

[22] Pedrazzoli P, Piralla A, Valentino F, Cinieri S, Baldanti F (2018). Update of the recommendations of the Italian Society of Medical Oncology on vaccination for seasonal influenza and pneumococcal infection in patients with cancer: focus on prevention of pneumonia. Eur J Cancer Care (Engl).

[23] Wong A, Marrie TJ, Garg S, Kellner JD, Tyrrell GJ, Group S (2010). Increased risk of invasive pneumococcal disease in haematological and solid-organ malignancies. Epidemiol Infect.

[24] Aliberti S, Ramirez JA (2014). Cardiac diseases complicating community-acquired pneumonia. Curr Opin Infect Dis.

[25] French survey on the immunization coverage, January 2011. Immunization coverage against seasonal influenza in target groups and measurement of vaccine efficacy. Immunization coverage with diphtheria-tetanus-polio (DTP) and pneumococcal vaccines in persons 65 years of age and older. https://www.santepubliquefrance.fr/maladies-et-traumatismes/maladies-a-prevention-vaccinale/diphterie/documents/rapport-synthese/enquete-nationale-de-couverture-vaccinale-france-janvier-2011.-couverture-vaccinale-contre-la-grippe-saisonniere-dans-les-groupes-cibles-et-mesur.

[26] Saba G, Andrade LF, Gaillat J, Bonnin P, Chidiac C, Illes HG, Laurichesse H, Messika J, Ricard JD, Detournay B (2018). Costs associated with community acquired pneumonia in France. Eur J Health Econ.

[27] Tiv M, Clinard F, Guthman J-P, Gavazzi G, Legris C, Tillier C, Vaux S, Lepoutre A, Aho LS, Fournel I (2010). Pneumococcal and tetanus vaccination coverage in residents of nursing homes for elderly people in Burgundy and Franche-Comté regions, France, 2009. Bull Epidemiol Hebdomadaire.

[28] Backhaus E, Berg S, Andersson R, Ockborn G, Malmstrom P, Dahl M, Nasic S, Trollfors B (2016). Epidemiology of invasive pneumococcal infections: manifestations, incidence and case fatality rate correlated to age, gender and risk factors. BMC Infect Dis.

[29] Loubet P, Kerneis S, Groh M, Loulergue P, Blanche P, Verger P, Launay O (2015). Attitude, knowledge and factors associated with influenza and pneumococcal vaccine uptake in a large cohort of patients with secondary immune deficiency. Vaccine.

[30] Sitte J, Frentiu E, Baumann C, Rousseau H, May T, Bronowicki JP, Peyrin-Biroulet L, Lopez A (2019). Vaccination for influenza and pneumococcus in patients with gastrointestinal cancer or inflammatory bowel disease: a prospective cohort study of methods for improving coverage. Aliment Pharmacol Ther.

[31] Danis K, Varon E, Lepoutre A, Janssen C, Forestier E, Epaulard O, N’Guyen Y, Labrunie A, Lanotte P, Gravet A (2019). Factors associated with severe nonmeningitis invasive pneumococcal disease in adults in France. Open Forum Infect Dis.

[32] Aronsky D, Haug PJ, Lagor C, Dean NC (2005). Accuracy of administrative data for identifying patients with pneumonia. Am J Med Qual.

[33] Lindenauer PK, Lagu T, Shieh MS, Pekow PS, Rothberg MB (2012). Association of diagnostic coding with trends in hospitalizations and mortality of patients with pneumonia, 2003–2009. JAMA.

[34] Said MA, Johnson HL, Nonyane BA, Deloria-Knoll M, O'Brien KL, Team AAPBS, Andreo F, Beovic B, Blanco S, Boersma WG, et al. Estimating the burden of pneumococcal pneumonia among adults: a systematic review and meta-analysis of diagnostic techniques. PloS One. 2013; 8(4):e60273. 10.1371/journal.pone.0060273 PMC3615022 23565216

[35] Smithee RB, Markus TM, Soda E, Grijalva CG, Xing W, Shang N, Griffin MR, Lessa FC (2020). Pneumonia hospitalization coding changes associated with transition from the 9th to 10th revision of international classification of diseases. Health Serv Res Manag Epidemiol.

[36] Yoon J, Chow A (2017). Comparing chronic condition rates using ICD-9 and ICD-10 in VA patients FY2014-2016. BMC Health Serv Res.

[37] Gisquet E, Aouba A, Aubry R, Jougla E, Rey G (2012). Where does one die in France ? Analysis of death certificates 1993–2008. Bull Epidemiol Hebdo.

