# Supplementary material for: The impact of comorbidities and their stacking on short- and long-term prognosis of patients over 50 with community-acquired pneumonia
Source: BMC Infect Dis. 2021 Sep 14;21:949. doi: 10.1186/s12879-021-06669-5 (PMC8442401; doi:10.1186/s12879-021-06669-5)
Supplement: Supplementary file 1 — Additional file 1: Figure S1. Patients flow chart. CAP: community-acquired pneumonia (all causes); P-CAP: community-acquired pneumococcal pneumonia; S. pneumoniae: Streptococcus pneumoniae; ICU: intensive care unit. The short-term deaths are those occurring during the hospital stay related to the CAP management, while the one-year in-hospital deaths are the in-hospital deaths occurring within the year following the initial hospital stay. Table S1. Comorbidities and other characteristics of patients with community-acquired pneumonia of all causes and of those with pneumococcal community-acquired pneumonia, according to the age groups. Table S2. Comorbidities or other characteristics that are risk factors for the admission in intensive care unit for patients with community-acquired pneumonia and those with pneumococcal community-acquired pneumonia, after adjustment on age and sex (Cox univariate model). Table S3. Comorbidity or other characteristics that are risk factors for the transfer in intensive care unit of patients > 50 years old hospitalized for community-acquired pneumonia overall, after adjustment on age and sex, overall and according to their age group (Cox univariate model). Table S4. In-hospital mortality of patients with community-acquired pneumonia according to the comorbidities categories, overall and according to their age group, during the initial hospital stay and during the subsequent hospital stays within the year following the CAP onset. Table S5. Comorbidity or other characteristics that are risk factors for the one-year in-hospital mortality for patients with community-acquired pneumonia and those with pneumococcal community-acquired pneumonia, after adjustment on age and sex (Cox univariate model). Table S6. Comorbidity or other characteristics that are risk factors for the one-year mortality of patients > 50 years old hospitalized for community-acquired pneumonia, after adjustment on age and sex, overall and according to their age group (Cox [file 12879_2021_6669_MOESM1_ESM.docx]

# Additional materials

**The impact of comorbidities and their stacking on short- and long-term prognosis of patients over 50 with community-acquired pneumonia**

E. Blanc^1^, G. Chaize^2^, S. Fievez^1^, C. Féger^3^, E. Herquelot^2^, A. Vainchtock^2^, JF. Timsit^4,5^, J. Gaillat^6^

## Methods:

## ****Definitions****

Two categories of comorbidities are commonly defined according to the magnitude of the associated risk of occurrence of pneumococcal disease in patients: at-risk (AR) comorbidities, in immunocompetent patients, or high risk (HR) comorbidities, mainly in immunocompromised patients [1, 2]. Thus, AR patients are immunocompetent patients who have at least one of the following chronic conditions: cardiac diseases (e.g., cardiac failure regardless of its origin, diseases of the cardiac valves, essential or secondary hypertension and related cardiac complications, other diseases of the heart, disorders of the heart rate, congenital heart disease, and complications of cardiac diseases), chronic lung disease (i.e., chronic respiratory insufficiency, emphysema, chronic obstructive pulmonary disease), severe asthma, diabetes mellitus, cerebrospinal fluid leak, cochlear implant, chronic liver disease (due to alcoholism or not), and renal insufficiency. HR patients are mostly immunocompromised patients with at least one of the following comorbidities: patients with asplenia or hyposplenia, primitive immune deficiency, human immunodeficiency virus (HIV) infection, nephrotic syndrome, patient with hematological malignancy or solid tumor requiring chemotherapy, solid organ transplant or hematopoietic stem cell transplant, or iatrogenic immunosuppression.

## ****Codes used for pneumonia diagnosis****

Detailed codes used for pneumonia diagnosis: A212 Pulmonary tularemia; A221 Lung anthrax; A370 ; Whooping cough caused by Bordetella pertussis; A371 Whooping cough caused by Bordetella parapertussis; A378 Whooping cough caused by other Bordetella species; A379 Whooping cough, unspecified; A420 Pulmonary actinomycosis; A430 Pulmonary nocardiosis ; A481 Legionnaires' disease (pulmonary); A70 Chlamydia psittaci infection; A78 Q fever; B012 Varicella pneumonia (J17.1 *); B052 Measles complicated by pneumonia (J17.1 *); B068 Rubella with other complications; B250 Cytomegalovirus pneumonia (J17.1 *); B371 Pulmonary candidiasis; B380 Acute pulmonary coccidioidomycosis; B382 Pulmonary coccidioidomycosis, unspecified; B390 Acute pulmonary histoplasmosis caused by Histoplasma capsulatum; B391 Chronic pulmonary histoplasmosis caused by Histoplasma capsulatum; B392 Pulmonary histoplasmosis caused by Histoplasma capsulatum, unspecified; B393 Histoplasmosis, disseminated caused by Histoplasma capsulatum; B394 Histoplasmosis caused by Histoplasma capsulatum, unspecified; B395 Histoplasmosis caused by Histoplasma duboisii; B399 Histoplasmosis, unspecified; B440 Invasive pulmonary aspergillosis; B441 Other pulmonary aspergillosis; B583 Pulmonary toxoplasmosis (J17.3 *); B59 Pneumocystosis; J100 Influenza with pneumonia, other influenza virus identified; J110 Influenza with pneumonia, unidentified virus; J120 Adenoviral pneumonia; J121 Respiratory syncytial virus [RSV] lung disease; J122 Pneumopathy caused by para-influenza virus; J123 Pneumonia caused by human metapneumovirus; J128 Other viral pneumonia; J129 Viral pneumonia, unspecified; D13 Pneumonia caused by Streptococcus pneumoniae; J14 Pulmonary disease caused by Haemophilus influenzae; J150 Pneumonia caused by Klebsiella pneumoniae; J151 Pneumonia caused by Pseudomonas; J152 Pneumonia caused by staphylococci; J153 Pneumonia caused by streptococci, group B; J154 Pneumonia caused by other streptococci; J155 Pneumonia caused by Escherichia coli; J156 Pneumonia caused by other aerobic Gram-negative bacteria; J157 Pneumonia caused by Mycoplasma pneumoniae; J158 Other bacterial pneumonia; J159 Bacterial pneumonia, unspecified; J160 Pneumonia caused by Chlamydia; J168 Pneumonia caused by other infectious microorganisms; J170 Pulmonary disease in bacterial diseases classified elsewhere; J171 Pulmonary disease in viral diseases classified elsewhere; J172 Pneumopathy during mycosis; J178 Pneumonia in other diseases classified elsewhere; J180 Bronchopneumopathy, unspecified; J181 Lobar pulmonary disease, unspecified; J188 Other pneumonia, unspecified microorganism; J189 Pneumopathy, unspecified; J690 Pneumonia due to food and vomiting; J850 Gangrene and lung necrosis if specified infectious; J851 Lung abscess with pneumonia.

Codes used for diagnosis of pneumococcal etiology: J13 Pneumonia caused by *Streptococcus pneumoniae*, and B953 Other diseases classified elsewhere caused by *Streptococcus pneumoniae*.

Codes used for secondary diagnosis of pneumonia: B206, B24+9, C340, C341, C342, C343, C349, C780, D022, D143, D381, I200, I200+0, I201, I209, I210, I2100, I21000, I2108, I211, I2110, I21100, I2118, I212, I2120, I21200, I2128, I2130, I21300, I2138, I2140, I21400, I2148, I219, I2190, I21900, I2198, I2200, I2208, I2210, I22800, I500, I501, I509, J09, J101, J108, J111, J118, J173, J182, J440, J441, J448, J449, J450, J451, J458, J459, J46, J679, J691, J698, J704, J80, J81, J840, J841, J849, J860, J869, J90, J91, J930, J931, J938, J939, J960*, J961*, J969, R042, R060, R071, R072, R074, R91, Z512.

## ****Codes used for the comorbidities****

Alcohol abuse: F10, and tobacco abuse: F17.

High-risk comorbidities:

HIV: B20 to B24, Z21; asplenia/hyposplenia: D56, D57, D73; immune disorders and auto-immune diseases: D80 to D84, D89, K50, K51, L40, L93, M05 to M09, M32, M45 to M49.

Solid tumors: C00 to C26, C30 to C34, C37 to C41, C43 to C58, C60 to C80, C97, D00 to D07, D09, D37 to D44, D48; malignant hemopathies: C81 to C86, C88, C90 to C96, D46, D47, D61.

Transplant: T86, Z94, Z95

At-risk comorbidities:

Diabetes mellitus: E10 to E14

Chronic cardiac diseases: I05 to I11, I13, I15, I20 to II25, I27, I30 to I32, I34 to I37, I40 to I49, I51, I52; cardiac insufficiency: I50.

Chronic respiratory diseases: J41 to J43, J47, J960 (if asthma is associated); including chronic obstructive bronchial disease: J44, asthma: J45 and J46, and respiratory insufficiency J961

Chronic liver diseases: K73, K75, K76; including liver failure: K72, and cirrhosis: K70 and K74.

Chronic renal diseases: I12, N03, N04, N08, N11, N14, to N16, N19; including chronic renal failure: N18, Z49, Z992

Figure S1 – Patients flow chart

CAP: community-acquired pneumonia (all causes); P-CAP: community-acquired pneumococcal pneumonia; *S. pneumoniae: Streptococcus pneumoniae*; ICU: intensive care unit. The short-term deaths are those occurring during the hospital stay related to the CAP management, while the one-year in-hospital deaths are the in-hospital deaths occurring within the year following the initial hospital stay.

***
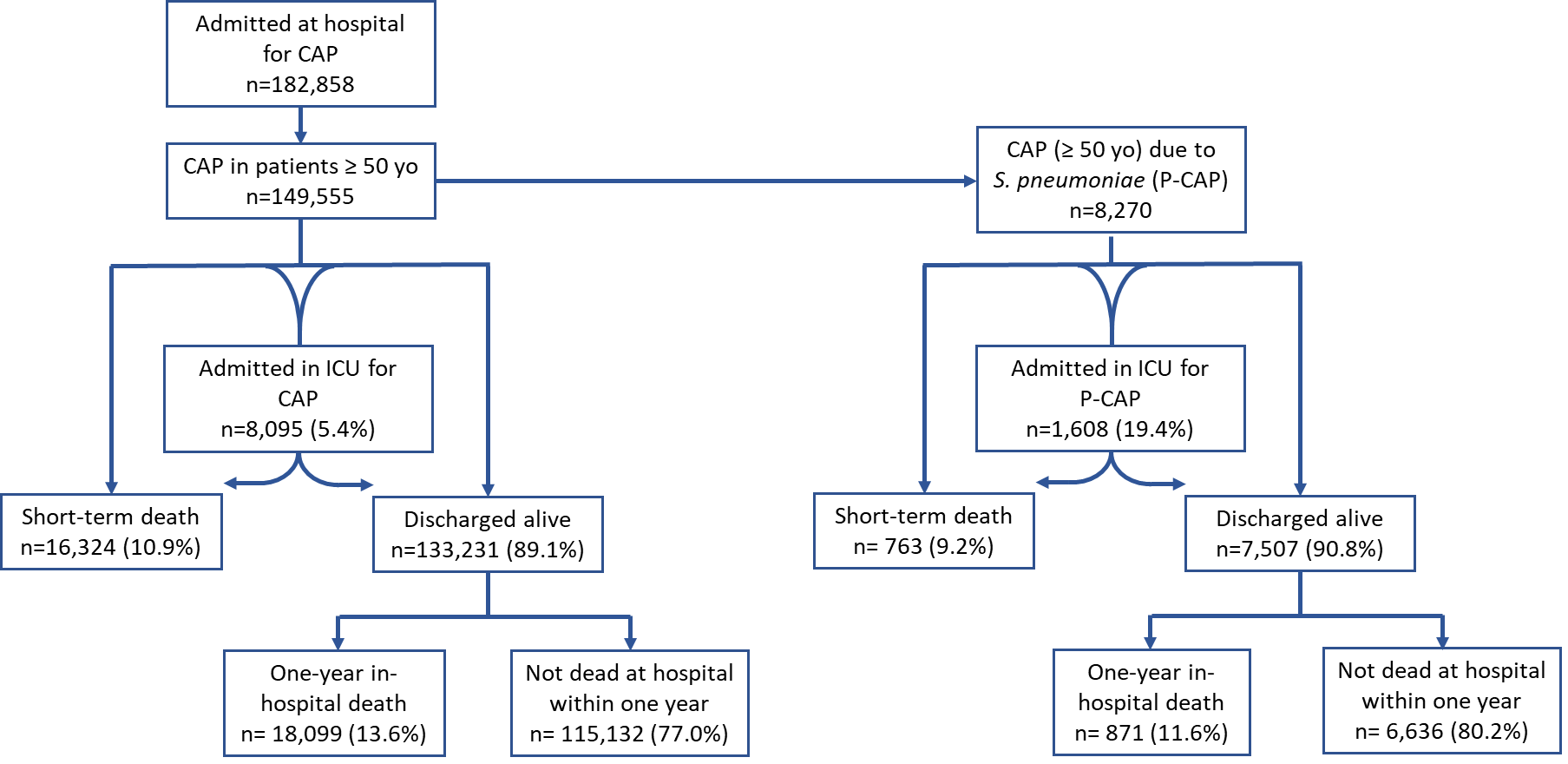
***

Table S1 – Comorbidities and other characteristics of patients with community-acquired pneumonia of all causes and of those with pneumococcal community-acquired pneumonia, according to the age groups

| **Variable** | **All CAP** | | | | **P-CAP** | | | |
| --- | --- | --- | --- | --- | --- | --- | --- | --- |
| **Age groups** | **50-69** | **70-89** | **>90** | **Overall** | **50-69** | **70-89** | **>90** | **Overall** |
| N | 37,807 | 85,159 | 26,589 | 149,555 | 3,257 | 4,114 | 899 | 8,270 |
| Sex (female) | 14,297 (37.82%) | 39,406 (46.27%) | 16,990 (63.90%) | 70,693 (47.27%) | 1,176 (36.11%) | 1,745 (42.42%) | 555 (61.74%) | 3,476 (42.03%) |
| Age (years) | 60.90 (+/-5.51) | 81.19 (+/-5.38) | 92.89 (+/-2.82) | 78.14 (+/-12.03) | 60.55 (+/-5.44) | 79.98 (+/-5.50) | 92.77 (+/-2.68) | 73.72 (+/-12.44) |
| Alcohol | 6,980 (18.46%) | 5,234 (6.15%) | 400 (1.50%) | 12,614 (8.43%) | 849 (26.07%) | 319 (7.75%) | 19 (2.11%) | 1,187 (14.35%) |
| Tobacco | 10,151 (26.85%) | 7,530 (8.84%) | 544 (2.05%) | 18,225 (12.19%) | 1,246 (38.26%) | 627 (15.24%) | 39 (4.34%) | 1,912 (23.12%) |
| **Comorbidities** |  |  |  |  |  |  |  |  |
| No comorbidity | 6,333 (16.75%) | 3,620 (4.25%) | 894 (3.36%) | 10,847 (7.25%) | 392 (12.04%) | 152 (3.69%) | 26 (2.89%) | 570 (6.89%) |
| Solely AR comorbidities | 17,337 (45.86%) | 51,807 (60.84%) | 18,977 (71.37%) | 88,121 (58.92%) | 1,687 (51.80%) | 2,409 (58.56%) | 642 (71.41%) | 4,738 (57.29%) |
| Solely HR comorbidities | 1,898 (5.02%) | 1,301 (1.53%) | 150 (0.56%) | 3,349 (2.24%) | 120 (3.68%) | 73 (1.77%) | 8 (0.89%) | 201 (2.43%) |
| At least one AR and one HR comorbidities | 12,239 (32.37%) | 28,431 (33.39%) | 6,568 (24.70%) | 47,238 (31.59%) | 1,058 (32.48%) | 1,480 (35.97%) | 223 (24.81%) | 2,761 (33.39%) |
| **HR comorbidities** |  |  |  |  |  |  |  |  |
| No HR comorbidity | 23,670 (62.61%) | 55,427 (65.09%) | 19,871 (74.73%) | 98,968 (66.17%) | 2,079 (63.83%) | 2,561 (62.25%) | 668 (74.30%) | 5,308 (64.18%) |
| HR comorbidities: n=1 | 11,432 (30.24%) | 25,203 (29.60%) | 6,035 (22.70%) | 42,670 (28.53%) | 944 (28.98%) | 1,280 (31.11%) | 199 (22.14%) | 2,423 (29.30%) |
| HR comorbidities: n=2-5 | 2,705 (7.15%) | 4,529 (5.32%) | 683 (2.57%) | 7,917 (5.29%) | 234 (7.18%) | 273 (6.64%) | 32 (3.56%) | 539 (6.52%) |
| No. of HR comorbidities | 0.47 (+/-0.69) | 0.41 (+/-0.62) | 0.28 (+/-0.51) | 0.40 (+/-0.62) | 0.46 (+/-0.70) | 0.45 (+/-0.64) | 0.30 (+/-0.54) | 0.44 (+/-0.66) |
| Solid tumors | 9,885 (26.15%) | 21,717 (25.50%) | 4,938 (18.57%) | 36,540 (24.43%) | 798 (24.50%) | 1,088 (26.45%) | 156 (17.35%) | 2,042 (24.69%) |
| Hematological malignancies | 2,491 (6.59%) | 6,736 (7.91%) | 1,466 (5.51%) | 10,693 (7.15%) | 226 (6.94%) | 412 (10.01%) | 67 (7.45%) | 705 (8.52%) |
| Autoimmune diseases | 1,860 (4.92%) | 4,113 (4.83%) | 758 (2.85%) | 6,731 (4.50%) | 144 (4.42%) | 212 (5.15%) | 28 (3.11%) | 384 (4.64%) |
| Primitive immunodeficiencies | 957 (2.53%) | 1,276 (1.50%) | 159 (0.60%) | 2,392 (1.60%) | 93 (2.86%) | 85 (2.07%) | 9 (1.00%) | 187 (2.26%) |
| Organ transplant | 1,567 (4.14%) | 654 (0.77%) | 77 (0.29%) | 2,298 (1.54%) | 113 (3.47%) | 38 (0.92%) | 2 (0.22%) | 153 (1.85%) |
| HIV | 578 (1.53%) | 118 (0.14%) | 2 (0.01%) | 698 (0.47%) | 81 (2.49%) | 10 (0.24%) | 0 (0.00%) | 91 (1.10%) |
| Asplenia - Hyposplenia | 266 (0.70%) | 324 (0.38%) | 46 (0.17%) | 636 (0.43%) | 31 (0.95%) | 13 (0.32%) | 4 (0.44%) | 48 (0.58%) |
| **AR comorbidities** |  |  |  |  |  |  |  |  |
| No AR comorbidity | 8,231 (21.77%) | 4,921 (5.78%) | 1,044 (3.93%) | 14,196 (9.49%) | 512 (15.72%) | 225 (5.47%) | 34 (3.78%) | 771 (9.32%) |
| AR comorbidities: n=1 | 10,547 (27.90%) | 16,646 (19.55%) | 4,957 (18.64%) | 32,150 (21.50%) | 855 (26.25%) | 796 (19.35%) | 168 (18.69%) | 1,819 (22.00%) |
| AR comorbidities: n=2 | 9,295 (24.59%) | 24,531 (28.81%) | 7,898 (29.70%) | 41,724 (27.90%) | 904 (27.76%) | 1,336 (32.47%) | 297 (33.04%) | 2,537 (30.68%) |
| AR comorbidities: n=3 | 5,802 (15.35%) | 20,976 (24.63%) | 7,048 (26.51%) | 33,826 (22.62%) | 606 (18.61%) | 1,026 (24.94%) | 230 (25.58%) | 1,862 (22.52%) |
| AR comorbidities: n=4-8 | 3,932 (10.40%) | 18,085 (21.24%) | 5,642 (21.22%) | 27,659 (18.49%) | 380 (11.67%) | 731 (17.77%) | 170 (18.91%) | 1,281 (15.49%) |
| No. of AR comorbidities | 1.69 (1.36) | 2.45 (1.35) | 2.51 (1.28) | 2.27 (1.38) | 1.89 (1.33) | 2.38 (1.28) | 2.43 (1.22) | 2.19 (1.32) |
| Chronic cardiac diseases | 21,630 (57.21%) | 70,749 (83.08%) | 23,323 (87.72%) | 115,702 (77.36%) | 1,915 (58.80%) | 3,407 (82.81%) | 796 (88.54%) | 6,118 (73.98%) |
| Malnutrition | 8,819 (23.33%) | 30,732 (36.09%) | 12,787 (48.09%) | 52,338 (35.00%) | 904 (27.76%) | 1,308 (31.79%) | 439 (48.83%) | 2,651 (32.06%) |
| Chronic respiratory diseases | 12,710 (33.62%) | 27,124 (31.85%) | 5,862 (22.05%) | 45,696 (30.55%) | 1,603 (49.22%) | 1,928 (46.86%) | 251 (27.92%) | 3,782 (45.73%) |
| Diabetes | 8,067 (21.34%) | 22,917 (26.91%) | 4,266 (16.04%) | 35,250 (23.57%) | 706 (21.68%) | 1,083 (26.32%) | 116 (12.90%) | 1,905 (23.04%) |
| Neurodegenerative diseases | 1,929 (5.10%) | 20,246 (23.77%) | 7,852 (29.53%) | 30,027 (20.08%) | 100 (3.07%) | 547 (13.30%) | 201 (22.36%) | 848 (10.25%) |
| Chronic renal diseases | 3,832 (10.14%) | 17,271 (20.28%) | 6,971 (26.22%) | 28,074 (18.77%) | 297 (9.12%) | 748 (18.18%) | 236 (26.25%) | 1,281 (15.49%) |
| Stroke | 3,367 (8.91%) | 16,187 (19.01%) | 5,051 (19.00%) | 24,605 (16.45%) | 215 (6.60%) | 525 (12.76%) | 134 (14.91%) | 874 (10.57%) |
| Chronic liver diseases | 3,466 (9.17%) | 3,631 (4.26%) | 502 (1.89%) | 7,599 (5.08%) | 405 (12.43%) | 232 (5.64%) | 14 (1.56%) | 651 (7.87%) |

CAP: community-acquired pneumonia; P-CAP: pneumococcal community-acquired pneumonia; HR: high-risk comorbidities (immunodepression, immunodeficiency, cancer); AR: at risk comorbidities (comorbidities at risk of pneumococcal CAP in immunocompetent patients); HIV: Human immunodeficiency virus; Q1-Q3: interquartile range. Qualitative variables are expressed as number of patients (percentages), quantitative variables as mean (SD).

Table S2 – Comorbidities or other characteristics that are risk factors for the admission in intensive care unit for patients with community-acquired pneumonia and those with pneumococcal community-acquired pneumonia, after adjustment on age and sex (Cox univariate model)

|  | **CAP** (n=149,555) | | | **P-CAP** (n=8,270) | | |
| --- | --- | --- | --- | --- | --- | --- |
| **Characteristics of the patients** |  | **HR [95% CI]** | **p value** |  | **HR [95% CI]** | **p value** |
| **Tobacco and/or alcohol** |  | 1.92 [1.82;2.02] | <.0001 |  | 1.54 [1.37;1.74] | <.0001 |
| Alcohol abuse | 12,614 (8.43%) | 1.97 [1.85;2.09] | <.0001 | 1,187 (14.35%) | 1.54 [1.33;1.78] | <.0001 |
| Tobacco abuse | 18,225 (12.19%) | 1.83 [1.73;1.93] | <.0001 | 1,912 (23.12%) | 1.40 [1.24;1.59] | <.0001 |
| **High-risk patients** |  | **1.26 [1.21;1.32]** | **<.0001** |  | **1.01 [0.90;1.13]** | **0.883** |
| No HR comorbidity | 98,968 (66.17%) | 1.00 (ref) | . | 5,308 (64.18%) | 1.00 (ref) | . |
| N = 1 HR comorbidity | 42,670 (28.53%) | 1.19 [1.14;1.26] | <.0001 | 2,423 (29.30%) | 1.01 [0.89;1.14] | 0.9029 |
| N ≥ 2 HR comorbidities | 7,917 (5.29%) | 1.62 [1.49;1.76] | <.0001 | 539 (6.52%) | 1.01 [0.81;1.26] | 0.909 |
| *Type of HR comorbidities* | 0.40 (+/-0.62) |  |  | 0.44 (+/-0.66) |  |  |
| Solid tumor | 36,540 (24.43%) | 1.13 [1.08;1.19] | <.0001 | 2,042 (24.69%) | 0.98 [0.86;1.11] | 0.7274 |
| Hematological malignancy | 10,693 (7.15%) | 1.41 [1.30;1.52] | <.0001 | 705 (8.52%) | 1.01 [0.82;1.23] | 0.9426 |
| Auto-immune disorders | 6,731 (4.50%) | 1.30 [1.18;1.43] | <.0001 | 384 (4.64%) | 1.07 [0.82;1.38] | 0.6323 |
| Primitive immune deficiency | 2,392 (1.60%) | 1.71 [1.49;1.95] | <.0001 | 187 (2.26%) | 1.00 [0.70;1.44] | 0.9878 |
| Organ transplant recipient | 2,298 (1.54%) | 1.57 [1.39;1.79] | <.0001 | 153 (1.85%) | 1.19 [0.83;1.71] | 0.3499 |
| HIV | 698 (0.47%) | 1.24 [0.98;1.56] | 0.0763 | 91 (1.10%) | 0.90 [0.55;1.47] | 0.6806 |
| Asplenia - hyposplenia | 636 (0.43%) | 1.51 [1.15;1.97] | 0.0026 | 48 (0.58%) | 0.91 [0.45;1.85] | 0.7891 |
| **At risk patients** |  | **2.53 [2.31;2.79]** | **<.0001** |  | **2.60 [2.06;3.29]** | **<.0001** |
| No AR comorbidity | 14,196 (9.49%) | 1.00 (ref) | . | 771 (9.32%) | 1.00 (ref) | . |
| N = 1 AR comorbidity | 32,150 (21.50%) | 1.84 [1.66;2.05] | <.0001 | 1,819 (22.00%) | 1.78 [1.37;2.31] | <.0001 |
| N = 2 AR comorbidities | 41,724 (27.90%) | 2.60 [2.35;2.87] | <.0001 | 2,537 (30.68%) | 2.74 [2.14;3.52] | <.0001 |
| N = 3 AR comorbidities | 33,826 (22.62%) | 3.11 [2.80;3.44] | <.0001 | 1,862 (22.52%) | 3.16 [2.44;4.08] | <.0001 |
| N ≥ 4 AR comorbidities | 27,659 (18.49%) | 3.15 [2.84;3.51] | <.0001 | 1,281 (15.49%) | 3.31 [2.53;4.32] | <.0001 |
| *Type of AR comorbidities* | 2.27 (1.38) |  |  | 2.19 (1.32) |  |  |
| Chronic cardiac diseases | 115,702 (77.36%) | 1.93 [1.82;2.05] | <.0001 | 6,118 (73.98%) | 2.02 [1.75;2.32] | <.0001 |
| Malnutrition | 52,338 (35.00%) | 1.26 [1.20;1.33] | <.0001 | 2,651 (32.06%) | 1.25 [1.11;1.41] | 0.0002 |
| Chronic respiratory diseases | 45,696 (30.55%) | 1.72 [1.65;1.81] | <.0001 | 3,782 (45.73%) | 1.37 [1.22;1.53] | <.0001 |
| Diabetes mellitus | 35,250 (23.57%) | 1.29 [1.23;1.36] | <.0001 | 1,905 (23.04%) | 1.23 [1.08;1.39] | 0.0017 |
| Neurodegenerative diseases | 30,027 (20.08%) | 0.49 [0.45;0.53] | <.0001 | 848 (10.25%) | 0.63 [0.50;0.80] | 0.0001 |
| Chronic kidney diseases | 28,074 (18.77%) | 1.36 [1.28;1.44] | <.0001 | 1,281 (15.49%) | 1.34 [1.15;1.57] | 0.0002 |
| Stroke | 24,605 (16.45%) | 0.89 [0.83;0.95] | 0.0005 | 874 (10.57%) | 1.09 [0.90;1.31] | 0.375 |
| Chronic liver diseases | 7,599 (5.08%) | 2.37 [2.21;2.54] | <.0001 | 651 (7.87%) | 2.01 [1.69;2.40] | <.0001 |

CAP: community-acquired pneumonia; P-CAP: pneumococcal community-acquired pneumonia; HR: high-risk comorbidities (immunodepression, immunodeficiency, cancer); AR: at risk comorbidities (comorbidities at risk of pneumococcal CAP in immunocompetent patients); HIV: Human immunodeficiency virus; Q1-Q3: interquartile range. OR: Odds ratio; CI: confidence interval

Table S3 – Comorbidity or other characteristics that are risk factors for the transfer in intensive care unit of patients > 50 years old hospitalized for community-acquired pneumonia overall, after adjustment on age and sex, overall and according to their age group (Cox univariate model)

|  | **All patients** | |  | | **Age group 50-69 y.o.** | | |  | **Age group 70-89 y.o.** | |  | **Age group ≥ 90 y.o.** | |
| --- | --- | --- | --- | --- | --- | --- | --- | --- | --- | --- | --- | --- | --- |
| **Characteristics of the patients** | **OR [95% CI]** | **p value** | |  | | **OR [95% CI]** | **p value** |  | **OR [95% CI]** | **p value** |  | **OR [95% CI]** | **p value** |
| **P-CAP** | 4.31 [4.05;4.58] | <.0001 | |  | | 3.95 [3.62;4.30] | <.0001 |  | 4.74 [4.34;5.18] | <.0001 |  | 4.05 [2.67;6.14] | <.0001 |
| **Tobacco and/or alcohol** | **1.92 [1.82;2.02]** | **<.0001** | |  | | **2.09 [1.95;2.24]** | **<.0001** |  | **1.75 [1.62;1.90]** | **<.0001** |  | **0.64 [0.28;1.45]** | **0.2859** |
| Alcohol abuse | 1.97 [1.85;2.09] | <.0001 | |  | | 2.30 [2.13;2.48] | <.0001 |  | 1.50 [1.35;1.68] | <.0001 |  | 0.25 [0.03;1.77] | 0.1639 |
| Tobacco abuse | 1.83 [1.73;1.93] | <.0001 | |  | | 1.75 [1.63;1.88] | <.0001 |  | 1.98 [1.82;2.17] | <.0001 |  | 1.05 [0.46;2.40] | 0.9003 |
| **HR comorbidities** | **1.26 [1.21;1.32]** | **<.0001** | |  | | **1.20 [1.12;1.29]** | **<.0001** |  | **1.33 [1.25;1.42]** | **<.0001** |  | **1.10 [0.82;1.49]** | **0.5199** |
| No HR comorbidity | 1.00 [.;.] | . | |  | | 1.00 [.;.] | . |  | 1.00 [.;.] | . |  | 1.00 [.;.] | . |
| N = 1 HR comorbidity | 1.19 [1.14;1.26] | <.0001 | |  | | 1.14 [1.06;1.23] | 0.0005 |  | 1.25 [1.16;1.34] | <.0001 |  | 1.15 [0.85;1.57] | 0.3655 |
| N ≥ 2 HR comorbidities | 1.62 [1.49;1.76] | <.0001 | |  | | 1.47 [1.31;1.66] | <.0001 |  | 1.81 [1.61;2.04] | <.0001 |  | 0.68 [0.25;1.85] | 0.4519 |
| *Type of HR comorbidities* |  |  | |  | |  |  |  |  |  |  |  |  |
| Solid tumor | 1.13 [1.08;1.19] | <.0001 | |  | | 1.06 [0.99;1.15] | 0.1118 |  | 1.20 [1.12;1.29] | <.0001 |  | 1.07 [0.76;1.49] | 0.706 |
| Hematological malignancy | 1.41 [1.30;1.52] | <.0001 | |  | | 1.34 [1.19;1.51] | <.0001 |  | 1.47 [1.33;1.63] | <.0001 |  | 1.06 [0.60;1.87] | 0.8314 |
| Auto-immune disorders | 1.30 [1.18;1.43] | <.0001 | |  | | 1.24 [1.07;1.44] | 0.0036 |  | 1.35 [1.19;1.54] | <.0001 |  | 1.03 [0.45;2.32] | 0.9478 |
| Primitive immune deficiency | 1.71 [1.49;1.95] | <.0001 | |  | | 1.62 [1.35;1.94] | <.0001 |  | 1.87 [1.53;2.28] | <.0001 |  |  |  |
| Organ transplant recipient | 1.57 [1.39;1.79] | <.0001 | |  | | 1.41 [1.22;1.64] | <.0001 |  | 2.30 [1.79;2.96] | <.0001 |  | 1.66 [0.23;12.03] | 0.6144 |
| HIV | 1.24 [0.98;1.56] | 0.0763 | |  | | 1.26 [0.99;1.62] | 0.0611 |  | 0.99 [0.44;2.25] | 0.9817 |  |  |  |
| Asplenia - hyposplenia | 1.51 [1.15;1.97] | 0.0026 | |  | | 1.88 [1.36;2.59] | 0.0001 |  | 1.01 [0.61;1.68] | 0.9546 |  |  |  |
| **AR comorbidities** | **2.53 [2.31;2.79]** | **<.0001** | |  | | **2.97 [2.65;3.33]** | **<.0001** |  | **1.72 [1.45;2.04]** | **<.0001** |  | **0.91 [0.46;1.78]** | **0.778** |
| No AR comorbidity | 1.00 [.;.] |  | |  | | 1.00 [.;.] | . |  | 1.00 [.;.] | . |  | 1.00 [.;.] | . |
| N = 1 AR comorbidity | 1.84 [1.66;2.05] | <.0001 | |  | | 1.92 [1.69;2.19] | <.0001 |  | 1.37 [1.14;1.65] | 0.0008 |  | 0.86 [0.42;1.80] | 0.6968 |
| N = 2 AR comorbidities | 2.60 [2.35;2.87] | <.0001 | |  | | 2.93 [2.58;3.32] | <.0001 |  | 1.72 [1.44;2.05] | <.0001 |  | 1.08 [0.54;2.16] | 0.8323 |
| N = 3 AR comorbidities | 3.11 [2.80;3.44] | <.0001 | |  | | 3.94 [3.46;4.49] | <.0001 |  | 1.93 [1.62;2.31] | <.0001 |  | 0.80 [0.39;1.63] | 0.539 |
| N ≥ 4 AR comorbidities | 3.15 [2.84;3.51] | <.0001 | |  | | 4.72 [4.12;5.41] | <.0001 |  | 1.80 [1.50;2.15] | <.0001 |  | 0.85 [0.41;1.75] | 0.6555 |
| *Type of AR comorbidities* |  |  | |  | |  |  |  |  |  |  |  |  |
| Chronic cardiac diseases | 1.93 [1.82;2.05] | <.0001 | |  | | 2.10 [1.95;2.27] | <.0001 |  | 1.67 [1.51;1.85] | <.0001 |  | 1.44 [0.90;2.31] | 0.1305 |
| Malnutrition | 1.26 [1.20;1.33] | <.0001 | |  | | 1.84 [1.71;1.97] | <.0001 |  | 0.97 [0.91;1.04] | 0.3922 |  | 0.76 [0.57;1.00] | 0.0468 |
| Chronic respiratory diseases | 1.72 [1.65;1.81] | <.0001 | |  | | 1.70 [1.59;1.82] | <.0001 |  | 1.77 [1.66;1.89] | <.0001 |  | 1.33 [0.99;1.80] | 0.0606 |
| Diabetes mellitus | 1.29 [1.23;1.36] | <.0001 | |  | | 1.34 [1.24;1.45] | <.0001 |  | 1.26 [1.17;1.34] | <.0001 |  | 1.14 [0.80;1.62] | 0.4649 |
| Neurodegenerative diseases | 0.49 [0.45;0.53] | <.0001 | |  | | 1.02 [0.88;1.18] | 0.822 |  | 0.39 [0.35;0.43] | <.0001 |  | 0.55 [0.39;0.78] | 0.0008 |
| Chronic kidney diseases | 1.36 [1.28;1.44] | <.0001 | |  | | 1.60 [1.45;1.76] | <.0001 |  | 1.26 [1.17;1.35] | <.0001 |  | 1.21 [0.90;1.62] | 0.2088 |
| Stroke | 0.89 [0.83;0.95] | 0.0005 | |  | | 1.22 [1.09;1.36] | 0.0004 |  | 0.76 [0.70;0.83] | <.0001 |  | 0.62 [0.41;0.92] | 0.0185 |
| Chronic liver diseases | 2.37 [2.21;2.54] | <.0001 | |  | | 2.44 [2.23;2.67] | <.0001 |  | 2.29 [2.05;2.57] | <.0001 |  | 1.66 [0.78;3.55] | 0.1908 |

CAP: community-acquired pneumonia; P-CAP: pneumococcal community-acquired pneumonia; HR: high-risk comorbidities (immunodepression, immunodeficiency, cancer); AR: at risk comorbidities (comorbidities at risk of pneumococcal CAP in immunocompetent patients); y.o.: year old; HIV: Human immunodeficiency virus; OR: Odds ratio; CI: confidence interval

Table S4 – In-hospital mortality of patients with community-acquired pneumonia according to the comorbidities categories, overall and according to their age group, during the initial hospital stay and during the subsequent hospital stays within the year following the CAP onset

| **Variable** | **In-hospital mortality during the initial hospital stay** | | | | **Subsequent in-hospital mortality within the following year** | | | |
| --- | --- | --- | --- | --- | --- | --- | --- | --- |
| **Age groups** | **50-69** | **70-89** | **>90** | **Overall** | **50-69** | **70-89** | **>90** | **Overall** |
| Only HR comorbidities | 99 (5.22%) | 98 (7.53%) | 26 (17.3%) | 223 (6.66%) | 321 (16.91) | 179 (13.8%) | 21 (14.0%) | 521 (15.6%) |
| Only AR comorbidities | 816 (4.71%) | 5,583 (10.8%) | 3,097 (16.3%) | 9,496 (10.8%) | 988 (5.70%) | 5,286 (10.2%) | 2,169 (11.4%) | 8,443 (9.58%) |
| HR and AR comorbidities | 1,239 (10.1%) | 3,688 (13.0%) | 1,164 (17.7%) | 6,091 (12.9%) | 2,666 (21.8%) | 5,135 (18.1%) | 956 (14.6%) | 8,757 (18.5%) |
| No comorbidity | 110 (1.74%) | 229 (6.33%) | 175 (19.6%) | 514 (4.74%) | 144 (2.27%) | 170 (4.70%) | 64 (7.16%) | 378 (3.48%) |
| Overall | 2,264 (5.99%) | 9,598 (11.3%) | 4,462 (16.8%) | 16,324 (10.9%) | 4,119 (10.9%) | 10,770 (12.7%) | 3,210 (12.1%) | 18,099 (12.1%) |

HR: high-risk comorbidities (immunodepression, immunodeficiency, cancer); AR: at risk comorbidities (comorbidities at risk of pneumococcal CAP in immunocompetent patients).

Table S5 – Comorbidity or other characteristics that are risk factors for the one-year in-hospital mortality for patients with community-acquired pneumonia and those with pneumococcal community-acquired pneumonia, after adjustment on age and sex (Cox univariate model)

|  | **CAP** (n=149,555) | | | **P-CAP** (n=8,270) | | |
| --- | --- | --- | --- | --- | --- | --- |
| **Characteristics of the patients** | **HR** | **[95% CI]** | **p value** | **HR** | **[95% CI]** | **p value** |
| **Tobacco and/or alcohol** | 1.189 | [1.155;1.225] | <.0001 | 1.204 | [1.074;1.350] | 0.0015 |
| Alcohol abuse | 1.275 | [1.228;1.323] | <.0001 | 1.302 | [1.132;1.498] | 0.0002 |
| Tobacco abuse | 1.168 | [1.130;1.208] | <.0001 | 1.145 | [1.014;1.292] | 0.0291 |
| **High-risk patients** | **1.706** | **[1.670;1.743]** | **<.0001** | **1.697** | **[1.539;1.872]** | **<.0001** |
| No HR comorbidity | 1 | (ref) | . | 1 | (ref) | . |
| N = 1 HR comorbidity | 1.686 | [1.648;1.724] | <.0001 | 1.73 | [1.561;1.917] | <.0001 |
| N ≥ 2 HR comorbidity | 1.823 | [1.749;1.901] | <.0001 | 1.547 | [1.284;1.864] | <.0001 |
| *Type of HR comorbidity* |  |  |  |  |  |  |
| Solid tumor | 1.797 | [1.757;1.838] | <.0001 | 1.832 | [1.653;2.029] | <.0001 |
| Hematological malignancy | 1.438 | [1.388;1.490] | <.0001 | 1.19 | [1.013;1.398] | 0.034 |
| Auto-immune disorders | 1.046 | [0.994;1.100] | 0.087 | 0.975 | [0.771;1.234] | 0.8351 |
| Primitive immune deficiency | 1.147 | [1.057;1.245] | 0.001 | 0.904 | [0.641;1.277] | 0.5682 |
| Organ transplant recipient | 1.162 | [1.065;1.268] | 0.0008 | 1.185 | [0.829;1.694] | 0.3528 |
| HIV | 0.847 | [0.705;1.019] | 0.0783 | 1.189 | [0.735;1.924] | 0.4814 |
| Asplenia - hyposplenia | 1.458 | [1.266;1.679] | <.0001 | 1.552 | [0.899;2.680] | 0.1147 |
| **At risk patients** |  |  |  |  |  |  |
| No AR comorbidity | 1 | (ref) | . | 1 | (ref) | . |
| N = 1 AR comorbidity | 1.491 | [1.411;1.576] | <.0001 | 1.588 | [1.222;2.063] | 0.0005 |
| N = 2 AR comorbidity | 1.829 | [1.734;1.929] | <.0001 | 1.97 | [1.533;2.533] | <.0001 |
| N = 3 AR comorbidity | 2.168 | [2.055;2.288] | <.0001 | 2.385 | [1.851;3.073] | <.0001 |
| N ≥ 4 AR comorbidity | 2.49 | [2.359;2.629] | <.0001 | 2.773 | [2.142;3.590] | <.0001 |
| *Type of AR comorbidity* |  |  |  |  |  |  |
| Chronic cardiac diseases | 1.311 | [1.273;1.349] | <.0001 | 1.535 | [1.346;1.750] | <.0001 |
| Malnutrition | 1.583 | [1.549;1.617] | <.0001 | 1.54 | [1.394;1.701] | <.0001 |
| Chronic respiratory diseases | 1.037 | [1.013;1.061] | 0.0021 | 0.924 | [0.837;1.020] | 0.1188 |
| Diabetes mellitus | 1.024 | [0.999;1.050] | 0.0576 | 1.114 | [0.995;1.247] | 0.0608 |
| Neurodegenerative diseases | 1.325 | [1.292;1.358] | <.0001 | 1.378 | [1.195;1.589] | <.0001 |
| Chronic kidney diseases | 1.283 | [1.251;1.316] | <.0001 | 1.337 | [1.184;1.510] | <.0001 |
| Stroke | 1.207 | [1.175;1.239] | <.0001 | 1.276 | [1.106;1.472] | 0.0008 |
| Chronic liver diseases | 1.488 | [1.426;1.554] | <.0001 | 1.859 | [1.594;2.168] | <.0001 |

CAP: community-acquired pneumonia; P-CAP: pneumococcal community-acquired pneumonia; HR: high-risk comorbidities (immunodepression, immunodeficiency, cancer); AR: at risk comorbidities (comorbidities at risk of pneumococcal CAP in immunocompetent patients); HIV: Human immunodeficiency virus; Q1-Q3: interquartile range. OR: Odds ratio; CI: confidence interval

Table S6 – Comorbidity or other characteristics that are risk factors for the one-year mortality of patients > 50 years old hospitalized for community-acquired pneumonia, after adjustment on age and sex, overall and according to their age group (Cox univariate model)

|  | **All patients** | | | **Age group 50-69 y.o.** | | | **Age group 70-89 y.o.** | | | **Age group ≥ 90 y.o.** | | | |
| --- | --- | --- | --- | --- | --- | --- | --- | --- | --- | --- | --- | --- | --- |
| **Characteristics of the patients** | **HR** | **[95% CI]** | **p value** | **HR** | **[95% CI]** | **p value** | **HR** | **[95% CI]** | **p value** | **HR** | **[95% CI]** | **p value** |  |
| **P-CAP** | 0.88 | [0.837;0.925] | <.0001 | 0.744 | [0.658;0.842] | <.0001 | 0.873 | [0.797;0.957] | 0.0038 | 1.053 | [0.877;1.263] | 0.58 |  |
| **Tobacco and/or alcohol** | **1.189** | **[1.155;1.225]** | **<.0001** | **1.445** | **[1.374;1.520]** | **<.0001** | **1.091** | **[1.049;1.135]** | **<.0001** | **0.928** | **[0.821;1.049]** | **0.2318** |  |
| Alcohol abuse | 1.275 | [1.228;1.323] | <.0001 | 1.516 | [1.432;1.605] | <.0001 | 1.144 | [1.085;1.206] | <.0001 | 0.898 | [0.747;1.079] | 0.2501 |  |
| Tobacco abuse | 1.168 | [1.130;1.208] | <.0001 | 1.356 | [1.287;1.429] | <.0001 | 1.07 | [1.022;1.121] | 0.0039 | 0.926 | [0.794;1.082] | 0.3338 |  |
| **HR comorbidities** | 1.706 | [1.670;1.743] | <.0001 | 3.888 | [3.688;4.098] | <.0001 | 1.525 | [1.483;1.568] | <.0001 | 1.139 | [1.083;1.197] | <.0001 |  |
| No HR comorbidity | 1 | (ref) | . | 1 | (ref) | . | 1 | (ref) | . | 1 | (ref) | . |  |
| N = 1 HR comorbidity | 1.686 | [1.648;1.724] | <.0001 | 3.984 | [3.774;4.207] | <.0001 | 1.495 | [1.452;1.539] | <.0001 | 1.135 | [1.078;1.196] | <.0001 |  |
| N ≥ 2 HR comorbidities | 1.823 | [1.749;1.901] | <.0001 | 3.489 | [3.210;3.792] | <.0001 | 1.694 | [1.605;1.787] | <.0001 | 1.168 | [1.023;1.334] | 0.0216 |  |
| *Type of HR comorbidity* |  |  |  |  |  |  |  |  |  |  |  |  |  |
| Solid tumor | 1.797 | [1.757;1.838] | <.0001 | 4.343 | [4.131;4.565] | <.0001 | 1.526 | [1.482;1.572] | <.0001 | 1.188 | [1.124;1.255] | <.0001 |  |
| Hematological malignancy | 1.438 | [1.388;1.490] | <.0001 | 1.706 | [1.574;1.849] | <.0001 | 1.48 | [1.417;1.547] | <.0001 | 1.067 | [0.971;1.173] | 0.1779 |  |
| Auto-immune disorders | 1.046 | [0.994;1.100] | 0.087 | 0.945 | [0.840;1.063] | 0.3487 | 1.092 | [1.027;1.162] | 0.0054 | 0.985 | [0.860;1.129] | 0.8296 |  |
| Primitive immune deficiency | 1.147 | [1.057;1.245] | 0.001 | 1.303 | [1.133;1.499] | 0.0002 | 1.147 | [1.032;1.274] | 0.011 | 0.712 | [0.513;0.988] | 0.042 |  |
| Organ transplant recipient | 1.162 | [1.065;1.268] | 0.0008 | 1.198 | [1.071;1.341] | 0.0016 | 1.126 | [0.972;1.305] | 0.1127 | 0.97 | [0.632;1.489] | 0.8905 |  |
| HIV | 0.847 | [0.705;1.019] | 0.0783 | 0.808 | [0.652;1.000] | 0.0504 | 0.919 | [0.635;1.332] | 0.6572 | 1.747 | [0.246;12.388] | 0.5768 |  |
| Asplenia - hyposplenia | 1.458 | [1.266;1.679] | <.0001 | 1.903 | [1.522;2.379] | <.0001 | 1.286 | [1.057;1.565] | 0.012 | 1.128 | [0.680;1.872] | 0.6418 |  |
| **AR comorbidities** | **1.925** | **[1.830;2.025]** | **<.0001** | **2.425** | **[2.238;2.627]** | **<.0001** | **1.867** | **[1.729;2.015]** | **<.0001** | **1.027** | **[0.913;1.156]** | **0.6593** |  |
| No AR comorbidity | 1 | (ref) | . | 1 | (ref) | . | 1 | (ref) | . | 1 | (ref) | . |  |
| N = 1 AR comorbidity | 1.491 | [1.411;1.576] | <.0001 | 1.808 | [1.652;1.980] | <.0001 | 1.339 | [1.232;1.456] | <.0001 | 0.927 | [0.815;1.053] | 0.2442 |  |
| N = 2 AR comorbidities | 1.829 | [1.734;1.929] | <.0001 | 2.346 | [2.146;2.564] | <.0001 | 1.704 | [1.573;1.846] | <.0001 | 0.968 | [0.856;1.095] | 0.6061 |  |
| N = 3 AR comorbidities | 2.168 | [2.055;2.288] | <.0001 | 2.995 | [2.731;3.286] | <.0001 | 2.051 | [1.894;2.222] | <.0001 | 1.058 | [0.935;1.197] | 0.3691 |  |
| N ≥ 4 AR comorbidities | 2.49 | [2.359;2.629] | <.0001 | 3.534 | [3.209;3.893] | <.0001 | 2.39 | [2.207;2.589] | <.0001 | 1.158 | [1.022;1.312] | 0.0209 |  |
| *Type of AR comorbidity* |  |  |  |  |  |  |  |  |  |  |  |  |  |
| Chronic cardiac diseases | 1.311 | [1.273;1.349] | <.0001 | 1.509 | [1.432;1.589] | <.0001 | 1.298 | [1.247;1.350] | <.0001 | 1.027 | [0.959;1.101] | 0.4427 |  |
| Malnutrition | 1.583 | [1.549;1.617] | <.0001 | 2.872 | [2.733;3.018] | <.0001 | 1.54 | [1.498;1.583] | <.0001 | 1.074 | [1.027;1.124] | 0.0017 |  |
| Chronic respiratory diseases | 1.037 | [1.013;1.061] | 0.0021 | 1.106 | [1.050;1.164] | 0.0001 | 1.029 | [0.999;1.059] | 0.0592 | 0.998 | [0.946;1.053] | 0.9522 |  |
| Diabetes mellitus | 1.024 | [0.999;1.050] | 0.0576 | 1.032 | [0.973;1.095] | 0.299 | 1.016 | [0.986;1.048] | 0.2995 | 1.047 | [0.986;1.111] | 0.1322 |  |
| Neurodegenerative diseases | 1.325 | [1.292;1.358] | <.0001 | 1.814 | [1.661;1.982] | <.0001 | 1.375 | [1.334;1.417] | <.0001 | 1.115 | [1.063;1.170] | <.0001 |  |
| Chronic kidney diseases | 1.283 | [1.251;1.316] | <.0001 | 1.312 | [1.219;1.412] | <.0001 | 1.327 | [1.285;1.370] | <.0001 | 1.174 | [1.117;1.233] | <.0001 |  |
| Stroke | 1.207 | [1.175;1.239] | <.0001 | 1.394 | [1.292;1.503] | <.0001 | 1.231 | [1.191;1.273] | <.0001 | 1.064 | [1.006;1.125] | 0.0298 |  |
| Chronic liver diseases | 1.488 | [1.426;1.554] | <.0001 | 1.786 | [1.666;1.914] | <.0001 | 1.375 | [1.295;1.459] | <.0001 | 1.142 | [0.979;1.332] | 0.0907 |  |

CAP: community-acquired pneumonia; P-CAP: pneumococcal community-acquired pneumonia; HR: high-risk comorbidities (immunodepression, immunodeficiency, cancer); AR: at risk comorbidities (comorbidities at risk of pneumococcal CAP in immunocompetent patients); HIV: Human immunodeficiency virus; Q1-Q3: interquartile range. OR: Odds ratio; CI: confidence interval

1. **Vaccination recommendations against pneumococcal infections of at-risk people** [<https://www.hcsp.fr/Explore.cgi/avisrapportsdomaine?clefr=355>]

2. **Use of 13-valent pneumococcal conjugate vaccine and 23-valent pneumococcal polysaccharide vaccine for adults with immunocompromising conditions: recommendations of the Advisory Committee on Immunization Practices (ACIP)**. *MMWR Morb Mortal Wkly Rep* 2012, **61**(40):816-819.
